# Magnetic field effects as a result of the radical pair mechanism are unlikely in redox enzymes

**DOI:** 10.1098/rsif.2014.1155

**Published:** 2015-02-06

**Authors:** Hanan L. Messiha, Thanyaporn Wongnate, Pimchai Chaiyen, Alex R. Jones, Nigel S. Scrutton

**Affiliations:** 1 Manchester Institute of Biotechnology, University of Manchester, Manchester, UK; 2 Faculty of Life Sciences, University of Manchester, Manchester, UK; 3 Photon Science Institute and School of Chemistry, University of Manchester, Manchester, UK; 4 Department of Biochemistry and Centre for Excellence in Protein Structure and Function, Faculty of Science, Mahidol University, Bangkok, Thailand

**Keywords:** magnetic field effects, radical pair mechanism, flavoproteins, hydride transfer, environmental magnetic fields

## Abstract

Environmental exposure to electromagnetic fields is potentially carcinogenic. The radical pair mechanism is considered the most feasible mechanism of interaction between weak magnetic fields encountered in our environment and biochemical systems. Radicals are abundant in biology, both as free radicals and reaction intermediates in enzyme mechanisms. The catalytic cycles of some flavin-dependent enzymes are either known or potentially involve radical pairs. Here, we have investigated the magnetic field sensitivity of a number of flavoenzymes with important cellular roles. We also investigated the magnetic field sensitivity of a model system involving stepwise reduction of a flavin analogue by a nicotinamide analogue—a reaction known to proceed via a radical pair. Under the experimental conditions used, magnetic field sensitivity was not observed in the reaction kinetics from stopped-flow measurements in any of the systems studied. Although widely implicated in radical pair chemistry, we conclude that thermally driven, flavoenzyme-catalysed reactions are unlikely to be influenced by exposure to external magnetic fields.

## 1. Introduction

There is continued public concern over the possible impact of environmental exposure to electromagnetic fields on human health [1]. Sources of magnetic fields (MFs) are pervasive in our environment from the generation and distribution of electricity, the rapidly expanding mobile telecommunications industry and technologies such as magnetic resonance imaging. Periodically, reports appear in the literature suggesting MF toxicity in humans [2,3]. Perhaps of greatest significance are the epidemiological data that show a moderate association between residential proximity to high-voltage power lines and childhood leukaemia [4–7]. These persistent observations made from across the world over several decades have compelled the International Agency for Research on Cancer (IARC) to categorize extremely low frequency MFs as ‘possibly carcinogenic to humans’ in their report (IARC 2002) to the World Health Organization [8]. However, in 2011, the IARC published a review of the evidence on health risks of magnetic field effects (MFEs) concluding that current evidence is inadequate to confirm the existence of health consequences from exposure to low level environmental MFs [9]. The current absence of an established mechanism of action means it is difficult to confirm environmental MFs as a disease agent.

The radical pair mechanism (RPM) provides a robust experimental and theoretical framework from which to rationalize the observation of MFEs in chemical reactions [10,11]. When two radicals are produced simultaneously, for example by electron transfer or bond homolysis, spin multiplicity is conserved, and the unpaired electron spins are initially correlated in either a singlet (S, anti-parallel ↑↓) or triplet (T, parallel ↑↑) configuration, depending on the multiplicity of the radical precursors. If each unpaired spin experiences a different local MF, under certain conditions, the S and T RP spin-states will interconvert. The extent or rate of interconversion can be influenced by MFs, including weak fields such as those typically encountered in our environment (∼µT) [12]. For MFEs to manifest in the rate or product yield of a chemical reaction, the spin-states of the RP intermediates must have different reactive fates.

Radicals are widespread in biology. Reactive free radicals can cause significant biological damage with potentially fatal consequences. However, radicals can also serve as transient reaction intermediates in numerous enzyme reaction cycles [13]. Moreover, they play a crucial role in activating various signalling pathways affecting gene expression and initiating cell death [14,15]. Attempts to measure MFEs and their rationalization in the context of the RPM in biochemical systems have a chequered history. Despite a number of reports from various enzyme systems, not all MFEs have been reproduced independently [16–21]. However, it seems increasingly likely that some animals use a photochemical RP reaction as at least one means by which to detect the earth's MF [22]. Flavoproteins related to DNA photolyases, the cryptochromes, are proposed to undergo magnetically sensitive electron transfer chemistry. MFEs have been recorded in members of the cryptochrome/photolyase family of proteins [23,24]. Cryptochrome RPs seem to fulfil the chemical, magnetic, kinetic, structural and dynamic requirements of radical pair-based magnetoreception [22,25,26]. Therefore, the RPM is positioned as the most plausible known mechanism for interaction of external MFs with biological reaction kinetics.

**Figure 1.**
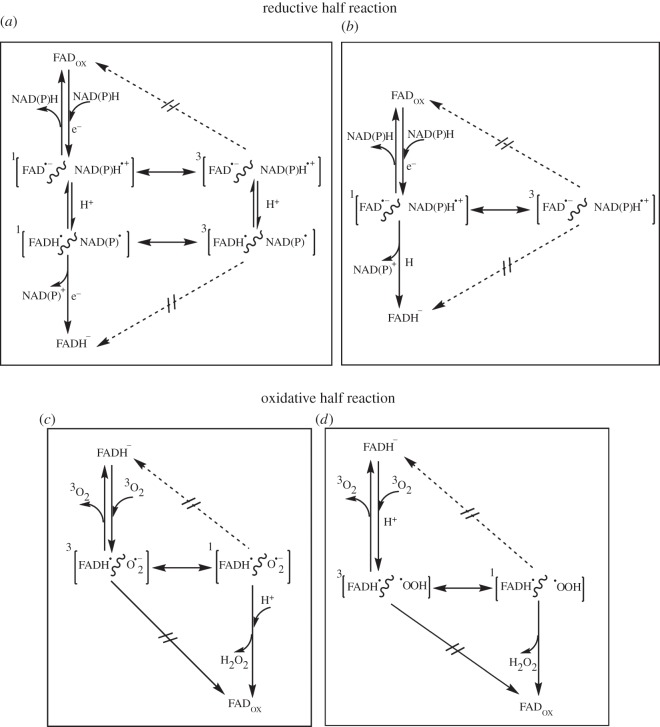
Possible radical pair reactions in redox reactions of flavoproteins. In the reductive half-reaction of flavoproteins, ‘hydride’ transfer from NAD(P)H to FAD_ox_ is represented by a putative stepwise mechanism [29]: (*a*) three steps (electron–proton–electron transfer); (*b*) two steps (electron–hydrogen transfer). Consequently, stepwise ‘hydride’ transfer can create charged [FAD**^•^**^−^/NAD(P)H**^•^**^+^] and neutral [FADH**^•^**/NAD(P)**^•^**] spin-correlated RPs. In (*a,b*), the T RP spin states are mostly non-productive—i.e. they can neither recombine nor proceed fully in the forward direction. (*c*) Electron transfer from the reduced flavin (e.g. FADH^−^) to O_2_ in monooxygenases and oxidases generates an [FADH**^•^**/O_2_**^•^**^−^] spin-correlated RP, which is followed by proton transfer to produce the oxidized flavin either directly (as in oxidases) or through the C4a-(hydro)peroxyflavin intermediate (as in monooxygenases, not shown). (*d*) Electron transfer from FADH^−^ to O_2_ in pyranose 2-oxidase is coupled with proton transfer from a conserved residue His548, creating [FADH**^•^**/**^•^**OOH] spin-correlated RPs. A C4a-(hydro)peroxyflavin intermediate (not shown for the sake of simplicity) is then formed and undergoes a unimolecular reaction to generate H_2_O_2_ and oxidized flavin [30]. In (*c,d*), only the T RP spin states can recombine, and only the S RP spin state can proceed in the forward direction.

Flavin-dependent enzymes are ubiquitous oxidoreductases, which play a fundamental role in biology catalysing a large variety reaction types. The isoalloxazine moiety of the flavin cofactor that acts as chromophore in cryptochromes can also undergo thermally driven redox chemistry. The different redox states of flavin confer catalytic function in a variety of one-electron and two-electron processes [27] and are therefore involved in a wide range of important biological processes such as energy production, oxidation/reduction, chromatin remodelling, DNA repair, apoptosis, protein folding, detoxification, neural development, biosynthesis, the circadian clock, photosynthesis, light emission and biodegradation [28]. In principle, various transient RP intermediates are possible during reactions catalysed by flavin-dependent enzymes (figure 1). Although reductive ‘hydride’ transfer is often treated as a concerted process, stepwise mechanisms have been observed in a number of small molecule model systems involving NADH and flavin analogues [31,32]. These proceed either via an electron–proton–electron transfer or electron–hydrogen transfer, with S-born ionic and neutral RPs possible [29]. We therefore hypothesize that RP intermediates are also possible in hydride transfer to fully oxidized flavin in the reductive half-reactions of flavoproteins (figure 1*a,b*). Here, only the S RP spin states can either recombine or proceed fully in the forward direction. Recombination of the geminate pair, hydrogen transfer to a flavin radical and electron transfer to a flavin radical all involve the combination of two unpaired spins to a S ground state and are therefore forbidden from the T RP spin states.

In the oxidative half-reaction, the first step of the reaction between reduced flavin and oxygen can involve an electron transfer from the reduced flavin to O_2_ to form a neutral flavin semiquinone/superoxide RP followed by a second electron and a proton transfer to form oxidized flavin and H_2_O_2_ (figure 1*c*). Alternatively, proton-coupled electron transfer from the reduced flavin to dioxygen to form a flavin semiquinone/hydroperoxy RP is possible (figure 1*d*), which also ultimately produces oxidized flavin and H_2_O_2_. Owing to the T ground state of molecular oxygen, the RP is T-born and its reactivity is highly spin-selective. Only the T RPs can recombine to regenerate the T ground state of O_2_, and only the S RPs can undergo the second electron transfer to give the S products [33].

For magnetically induced changes to RP reaction kinetics to have a meaningful physiological impact, the spin dynamics must be able to influence either the rate of enzyme turnover or resultant-free radical concentration. The putative RP intermediates in figure 1 are likely to have lifetimes that are fleeting from a biological standpoint (i.e. not gated by slow protein motions), and as such the spin dynamics are more likely to impact the reaction kinetics. We have therefore targeted systems (table 1) where ‘hydride’ transfer might reasonably proceed via the RP intermediates proposed in figure 1*a*,*b*. These include mammalian flavoprotein enzymes that impact important physiological processes and where we can measure accurately the pre-steady-state kinetics of the reductive half-reaction in the presence of an applied MF. It might be tempting to also consider diflavin enzyme systems where stable, very long-lived (ms–s) radicals are produced [34,35]. However, in such cases, the overall reaction rate might not be limited by the spin chemistry but by some influence of the protein (e.g. domain motions) [36]. We have also probed the oxidative reaction of pyranose 2-oxidase (P2O) from *Trametes multicolour*, where proton-coupled electron transfer from the reduced flavin to dioxygen forms a flavin semiquinone/hydroperoxy RP (figure 1*d*). A C4a-hydroperoxyflavin intermediate is then formed which decays into fully oxidized flavin and H_2_O_2_ [30].

**Table 1.** List of flavoproteins examined for MF sensitivity.

‘hydride’ transfer reactions in selected flavoproteins	physiological function
diflavin enzyme systems	
human cytochrome P450 reductase (CPR)—full-length enzyme and isolated FAD domain	homeostasis, drug detoxification, and xenobiotic metabolism
rat calmodulin-bound nitric oxide synthase (nNOS)—full-length enzyme and isolated FAD domain	cellular signalling
simple enzyme systems (one flavin)	
human apoptosis-inducing factor1 (AIF1)	apoptosis
human apoptosis-inducing factor 2 (AMID)	apoptosis
human soluble NADH-cytochrome *b*_5_ reductase (cyt *b*_5_ reductase)	methemoglobin reduction
human dihydroorotate dehydrogenase (DHODH)	pyrimidine biosynthesis
oxidative half-reaction of flavoproteins	
pyranose 2-oxidase (P2O) from *Trametes multicolor*	

## 2. Methods

Expression constructs for the targeted enzymes (those listed in table 1 alongside P2O from *T. multicolour*) were either available in our laboratories or obtained by commercial gene synthesis at Eurofins MWG Operon applying codon optimization for *E. coli* expression. Protein expression and purification followed published or modified protocols (for full Details, see the Experimental details section in the electronic supplementary material). The stepwise ‘hydride’-transfer during the reduction of the *p*-quinone (*p*-Q) derivative 1-(*p*-tolysulfinyl)-2,5-benzoquinone (TolSQ) by the NADH analogue 10-methyl-9,10-dihydroacridine (AcrH_2_) was also tested for potential MF sensitivity. All chemicals and reagents used were of analytical grade. AcrH_2_ and TolSQ were synthesized by NewChem Technologies Limited, UK (more than 98% purity).

The effect of MF exposure on the reaction kinetics was assessed from single-wavelength measurements using an Applied Photophysics SX.18MV-R stopped-flow spectrophotometer modified for MFE studies (MF effect stopped-flow spectrophotometer, MFESFS) as described in full elsewhere [17]. The applied magnetic field was generated by pairs of triple-coated neodymium–iron–boron (NdFeB) permanent magnets (from e-Magnets UK) that can be attached to or detached from to the MFESFS to record field-on and field-off measurements. A range of MFs (approx. 10–160 mT) was applied to the stopped-flow reaction cell. The set-up used is similar to that used previously to record MFEs in continuous wave-photolysis MFE studies [37].

All measurements were performed inside an anaerobic glovebox (Belle Technology UK Ltd); buffers and solutions were degassed by bubbling with nitrogen. For most enzyme systems examined for MFEs in the reductive half-reactions, the bleach of the flavin peak at approximately 450 nm (flavin reduction) was monitored to give direct access to ‘hydride’-transfer rates unless otherwise stated. NAD(P)H is the electron donor in most systems with the exception of DHODH where dihydroorotate (DHO) is the electron donor. Under all conditions, the electron donor concentration was chosen to saturate the enzyme active sites (i.e. at concentrations more than 10-fold the apparent dissociation constant for the enzyme–coenzyme or enzyme–substrate complex). In the reduction of TolSQ by AcrH_2_, the formation and decay of the AcrH_2_^•+^ species was monitored at 640 nm [32,38]. The formation and decay of the flavin–hydroperoxide intermediate during the oxidative reaction of P2O was followed at 395 nm [39]. Conditions specific to each enzyme system and data fitting are contained in the Experimental Details section in the electronic supplementary material.

For all measurements, the first 5–7 stopped-flow shots were discarded to ensure the acquisition of reproducible data. A homogeneous MF was applied to the sample position throughout the data acquisition period by placing pairs of permanent magnets either side of the reaction cell in unoccupied light-guide ports as described previously [37]. The MF dependence of the reaction kinetics of interest was investigated for each system using a broad range of moderate MFs (11.1–163.1 mT comprising 15 experiment sets). This approach allows us to confirm any magnetic perturbation as being a result of the RPM by observation of a saturation of the MFEs as a result of the Zeeman interaction (for separated RPs), or a peak in the MFE as a result of S/T level crossing (for RPs with a non-negligible exchange interaction) [10]. Each dataset comprised 12 field-off/field-on data acquisition pairs (the order of which was randomized). The rate of the reaction of interest (*k*) was determined for the field-on and field-off measurements as *k*_+MF_ and *k*_−MF_, respectively. The MFE was quantified after kinetic fitting as relative rates (*k*_+MF_/*k*_−MF_). In systems where the complexity of data analysis precluded the calculation of relative rates, difference traces (+MF data subtracting –MF data) were obtained. For more details, refer to the Experimental Details section in the electronic supplementary material.

## 3. Results and discussion

### 3.1. ‘Hydride’ transfer in diflavin oxidoreductase enzymes

**Figure 2.**
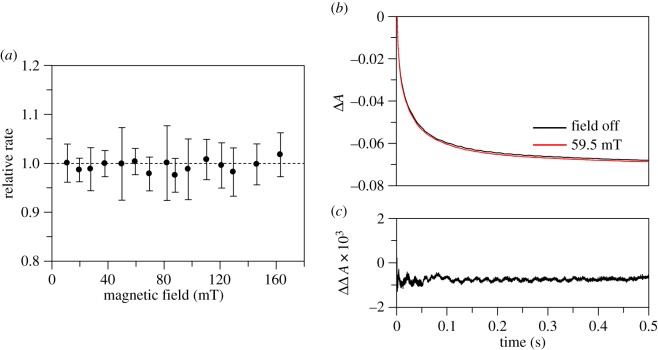
Influence of MFs on the kinetics of ‘hydride’ transfer in full-length diflavin enzymes. (*a*) MF dependence of the ‘hydride’ transfer rate in human full-length CPR quantified as a relative rate (*k*_+MF_/*k*_−MF_). The data points are the mean of 12 data acquisition pairs (−MF and +MF), and the error bars represent the standard deviation for each calculated relative rate. From the kinetic analysis, *k*_obs1_ is the ‘hydride’ transfer rate of interest (electronic supplementary material, figure S3 for more details). (*b*) Overlaid traces representing ‘hydride’ transfer reaction in full-length calmodulin-bound rat nNOS acquired at 0 mT (black) and 59.5 mT (red). (*c*) The corresponding difference trace where the data from 0 mT have been subtracted from those at 59.5 mT. The data represent examples of the data collected and similar results were also obtained at various magnetic field strengths (electronic supplementary material, figure S5). Any minor displacement observable in (*b*) and (*c*) is owing to experimental error, and the change in amplitude does not follow a meaningful trend with increasing MF (electronic supplementary material, figure S5). (Online version in colour.)

The mammalian NADPH-dependent diflavin oxidoreductases are redox enzymes with multiple domains and cofactors. Their complex reaction mechanisms are initiated by the transfer of two reductive equivalents from NADPH to flavin adenine dinucleotide (FAD) in the form of a ‘hydride’. Subsequent long-range, interdomain electron transfer between FAD and flavin mononucleotide (FMN) generates di-radical flavin semiquinone states that form/decay on the (ms–s) timescale. Electron transfer to downstream partner proteins completes the catalytic cycle [35,40]. We have focused on two important mammalian examples: human cytochrome P450 reductase (CPR), which plays a crucial role in homeostasis [41], drug detoxification and xenobiotic metabolism [42,43]; and rat neuronal nitric oxide synthase (nNOS), which is a key enzyme in cellular signalling [44,45]. Initially, the full-length enzymes were investigated by stopped-flow for potential MF sensitivity of the ‘hydride’ transfer kinetics. In human full-length CPR, reduction of FAD by NADPH is accompanied by a decrease in absorbance at 450 nm. This also leads to the accumulation of a blue di-semiquinoid species of the reductase (increase in absorbance at 600 nm), indicating rapid transfer of one electron to the FMN domain. The di-semiquinoid species decays on transfer of a second ‘hydride’, this time from FAD to FMN. Example kinetic traces at 450 and 600 nm, their fits and traces in the presence and absence of a 59.5 mT MF are displayed in the electronic supplementary material, figures S3 and S4. The MF dependence of the ‘hydride’ transfer relative rate in full-length human CPR is illustrated in figure 2*a* showing no significant MFE under the conditions used.

The reduction of calmodulin-bound rat nNOS reductase by NADPH was followed by stopped-flow at 458 nm as previously described by Knight & Scrutton [35]. The kinetics of this reaction are complex, precluding meaningful kinetic fitting. Potential MF sensitivity of the ‘hydride’ transfer kinetics was therefore analysed as difference traces at various MF strengths. Figure 2*b*,*c* illustrates no differences in kinetic traces by the application of 59.5 mT MF as an example. Similar results were obtained on applying MFs between 11.1 and 163.1 mT (12 pairs of measurements per field point; data are displayed in the electronic supplementary material, figure S5).

**Figure 3.**
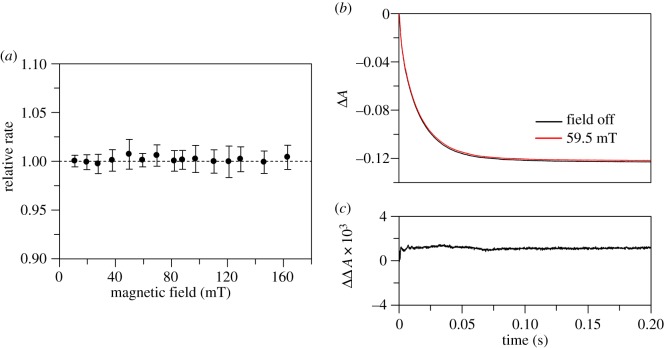
Influence of MFs on the kinetics of ‘hydride’ transfer in isolated FAD domains of diflavin enzymes. (*a*) MF dependence of the ‘hydride’ transfer rate in human full-length CPR quantified as a relative rate (*k*_+MF_/*k*_−MF_). The data points are the mean of 12 data acquisition pairs (−MF and +MF), and the error bars represent the standard deviation for each calculated relative rate. From the kinetic analysis, the ‘hydride’ transfer rate is *k*_obs2_ (electronic supplementary material, figure S6). (*b*) Overlaid traces representing ‘hydride’ transfer reaction in isolated FAD domain of rat nNOS acquired at 0 (black) and 59.5 mT (red). (*c*) The corresponding difference trace where the data from 0 mT have been subtracted from those at 59.5 mT. The data represent examples of the data collected, and similar results were also obtained at various MF strengths (electronic supplementary material, figure S8). Any minor displacement observable in (*b*) and (*c*) is owing to experimental error, and the change in amplitude does not follow a meaningful trend with increasing MF (electronic supplementary material, figure S8). (Online version in colour.)

These data suggest that neither human CPR nor rat nNOS in their natural state are likely to be carriers of mammalian MF sensitivity. However, the lack of an observable MFE is not diagnostic of the absence of RP intermediates. For example, the reaction catalysed by coenzyme B_12_-dependent ethanolamine ammonia lyase (EAL) is known to proceed via a cob(II)almin/5′-deoxyadenosyl radical pair generated by Co–C bond homolysis upon substrate binding. The reaction dynamics of the same RP generated by photolysis of free B_12_ (AdoCbl) are magnetically sensitive, and the magnitude of the MFE is enhanced by binding to the protein in the absence of substrate because of the active site acting as a RP ‘cage’ [19,37,46]. However, when the radicals are generated thermally by substrate binding to EAL, all magnetic-sensitivity is removed from the pre-steady-state kinetics. For MFEs to manifest, the extent of RP recombination needs to be significant. This does not seem to be the case in EAL, where the Co–C bond homolysis is kinetically coupled to subsequent H abstraction from the substrate. This quenches the 5′-deoxyadenosyl radical, limits recombination and removes the MFE [19,37]. A similar case arises with MFEs in photosynthetic reaction centres, which are not observed unless the electron transfer chain is blocked, allowing recombination [47]. By analogy, the rapid electron transfer between FAD and FMN that follows reduction of FAD by NADPH in CPR and NOS could limit the recombination of radicals formed during a stepwise ‘hydride’ transfer. Although a concerted transfer might explain the results in figure 3, we must first rule out the possible confounding effect of radical quenching.

We therefore investigated isolated FAD domains of CPR and nNOS to allow decoupling of the ‘hydride’ transfer step from the downstream chemistry. Measuring the kinetics of FAD reduction in the FAD domain of human CPR at 450 nm and 25°C as previously reported in Gutierrez *et al*. [34] does not fully resolve the early events. Reduction of the isolated FAD domain occurs in three kinetically resolvable steps. The first step represents a rapid formation of a charge-transfer species between oxidized FAD and NADPH that is too fast to be analysed by stopped-flow measurements. This is followed by isomerization to a second charge-transfer species, and the third step is ‘hydride’ transfer from NADPH to FAD [34]. We therefore conducted stopped-flow measurements at 10°C, which allowed for more accurate kinetic fitting of the data. (‘hydride’ transfer rate [*k*_obs2_] of 1.6 s^−1^ at 10°C, electronic supplementary material, figure S6). Again, the MF dependence showed no significant MFEs on ‘hydride’ transfer for the FAD domain of human CPR (figure 3*a*). Difference traces measured at 59.5 mT are illustrated as an example in the electronic supplementary material, figure S7. Similarly, difference traces from measurements made at 454 nm for the FAD domain of nNOS showed no MFE (e.g. traces at 59.5 mT, figure 3*b*,*c*). Data collected at other MFs can be found in the electronic supplementary material, figure S8. It therefore seems unlikely that the reductive chemistry in mammalian diflavin oxidoreductase enzymes such as CPR and nNOS, whether full-length or truncated, are magnetically sensitive as a result of RP chemistry.

### 3.2. ‘Hydride’ transfer in single-site flavoenzymes

In case truncation of diflavin oxidoreductases somehow influences the nature (stepwise or concerted) of the ‘hydride’ transfer mechanism, our investigations were extended to a number of ‘hydride’ transfer reactions catalysed by more flavoenzymes that contain only a single active site in their native state (i.e. with one flavin cofactor).

Apoptosis-inducing factor 1 (AIF1) is both a crucial early effector of apoptosis [48], and has NADH- and FAD-dependent oxidase activity, participating in cellular redox metabolism and mitochondrial bioenergetics [49]. There is evidence to suggest a connection between oxidase and apoptogenic functions of AIF1 [50] and that both are redox-controlled [51,52]. The AIF-homologous mitochondrion-associated inducer of death (AIF-M2 or AMID) has sequence similarities to AIF1. It has NAD(P)H and 6-hydroxy-FAD-dependent oxidase activity, catalysing the reduction of cytochrome *c* and other electron acceptors including molecular oxygen. There is a link between this redox activity and DNA binding that likely impacts its role in cellular apoptosis [53,54].

NADH-cytochrome *b_5_* reductase, a FAD-containing oxidoreductase, has two forms and catalyses the transfer of electrons from NADH to the haemoprotein cytochrome *b*_5_ [55]. In erythrocytes, the enzyme exists as a soluble form that catalyses the reduction of methemoglobin to haemoglobin, thus regulating methemoglobin concentrations [56]. Dihydroorotate dehydrogenase (DHODH) is a mitochondrial flavopotein that catalyses the ubiquinone-mediated oxidation of dihydroorotate to orotate, the fourth step in the de novo pyrimidine biosynthesis of DNA and RNA production [57]. It is a target for the treatment of cancer, rheumatoid arthritis and many other autoimmune diseases [58–60], and inhibition results in accumulation of the p53 tumour suppressor and apoptosis [61].

**Figure 4.**
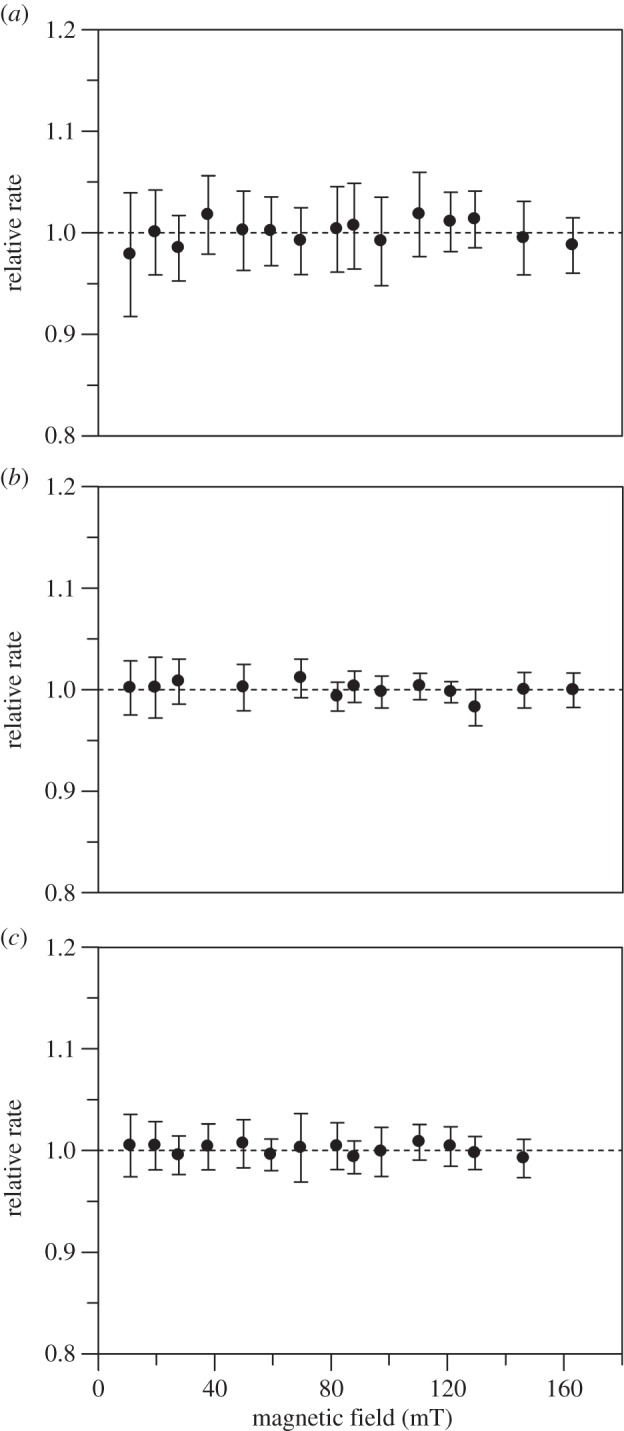
MF dependence of the kinetics of ‘hydride’ transfer in human monoflavin enzymes quantified as a relative rate (*k*_+MF_/*k*_−MF_). In all panels, the data points are the mean of 12 data acquisition pairs (−MF and +MF_on_), and the error bars represent the standard deviation for each calculated relative rate. (*a*) Human AIF1. From the kinetic analysis, the ‘hydride’ transfer rate is *k*_obs1_ (electronic supplementary material, figure S9). (*b*) Human cyt *b*_5_ reductase. From the kinetic analysis, the ‘hydride’ transfer rate is *k*_obs_ (electronic supplementary material, figure S11). (*c*) Human DHODH. From the kinetic analysis, the ‘hydride’ transfer rate is *k*_obs1_ (electronic supplementary material, figure S13).

**Figure 5.**
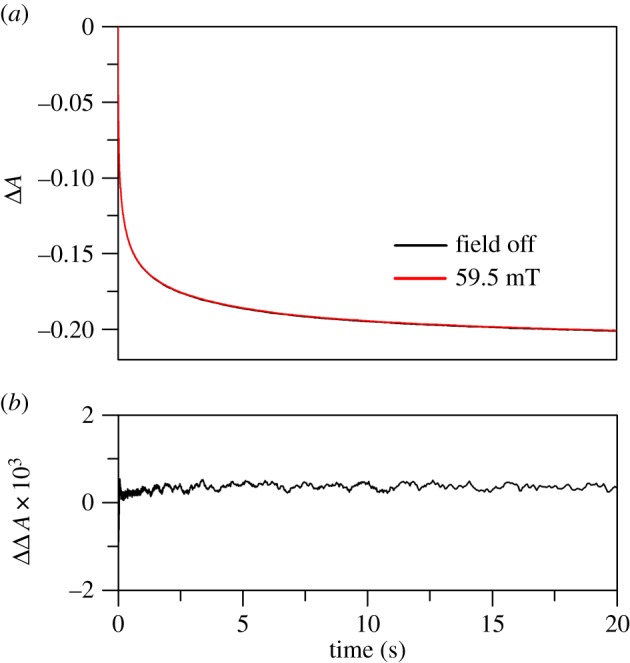
Influence of MFs on the kinetics of ‘hydride’ transfer reaction in human AMID. (*a*) Overlaid kinetic traces acquired at 0 (black) and 59.5 mT (red). (*b*) The corresponding difference trace where the data from 0 mT have been subtracted from those at 59.5 mT. The data represent examples of the data collected, and similar results were also obtained at various MF strengths (electronic supplementary material, figure S15). Any minor displacement observable in (*b*) and (*c*) is owing to experimental error, and the change in amplitude does not follow a meaningful trend with increasing MF (electronic supplementary material, figure S15). (Online version in colour.)

In all these monoflavin enzyme systems, no MFEs were observed under the conditions used (figures 4 and 5). The MF dependence of ‘hydride’ transfer rates in human AIF1, soluble cyt*b*_5_ reductase and DHODH are illustrated in figure 4*a*–*c*, respectively. In human AMID, the ‘hydride’ transfer reaction kinetic transients were complex, and therefore the influence of MFs was analysed using difference traces (figure 5 as an example recorded at a MF of 59.5 mT). Further details demonstrating kinetic traces, their fits and further difference traces can be found in the electronic supplementary material (figures S9 and S10 for AIF1; figures S11 and S12 for soluble cytb_5_ reductase, figures S13 and S14 for DHODH and figure S15 for AMID).

Despite the fact that RP intermediates are known to be possible as a result of stepwise ‘hydride’ transfer [29,31,32], no MFEs were observed for the ‘hydride’ transfer kinetics of the flavoproteins examined under the conditions used (figures 2–5). Two alternative explanations are therefore possible for enzymatic ‘hydride’ transfer: (i) it is predominantly a concerted process; (ii) stepwise transfer via radicals can happen, but the conditions for magnetic sensitivity are not met. We were able to account for one possible quenching route of radicals in diflavin oxidoreductases by isolating the FAD domain. However, we are unable to exclude the possibility of other influencing factors. In context, effects from rapid spin relaxation seem unlikely. Moreover, if the radicals of the pair are held sufficiently close that the exchange interaction between the unpaired spins precludes spin-state mixing, one might still anticipate a level crossing MFE within the MF range investigated. That said, alternative quenching channels are still possible, and spin dynamics might not influence measurable rates.

### 3.3. Stepwise ‘hydride’ transfer in the reduction of TolSQ by AcrH_2_

**Figure 6.**
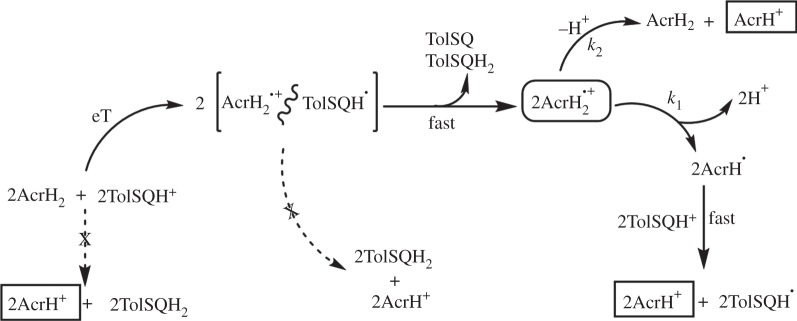
Mechanism of reduction of TolSQH^+^ by AcrH_2_ (adapted from Yuasa *et al*. [38]). eT, electron transfer.

**Figure 7.**
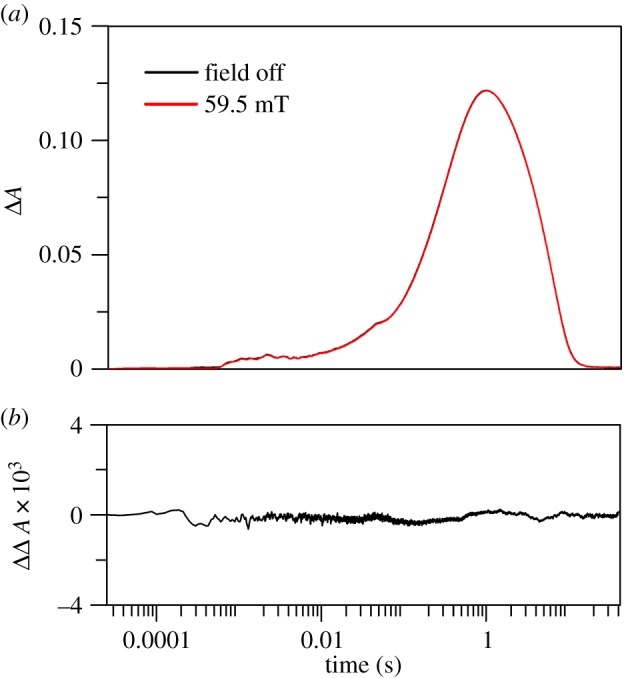
Influence of MFs on the kinetics of stepwise ‘hydride’ transfer during the reduction of TolSQ by AcrH_2_. (*a*) Overlaid absorbance traces recorded on a logarithmic time scale at 640 nm, acquired at 0 (black) and at 59.5 mT (red) for the reduction of TolSQ by AcrH_2_. (*b*) The corresponding difference trace where the data from 0 mT have been subtracted from those at 59.5 mT. The traces record the formation and decay of the AcrH_2_^+•^ species. The data shown are examples for illustration, and similar results were obtained at various MF strengths (electronic supplementary material, figure S16). (Online version in colour.)

To explore further the influence of reaction conditions on the magnetic sensitivity of RP reactions, we investigated an enzyme-free model system known to transfer a ‘hydride’ by a sequential mechanism. During the reduction of the protonated *p*-quinone (*p*-Q) derivative 1-(*p*-tolysulfinyl)-2,5-benzoquinone (TolSQH^+^) by the NADH analogue 10-methyl-9,10-dihydroacridine (AcrH_2_), an initial electron transfer (eT) occurs in preference to concerted ‘hydride’ transfer in the presence of perchloric acid (HClO_4_) [32,38]. A further benefit of such a system is that it excludes the possibility of protein dynamics gating the rate limiting chemistry. We monitored the formation and decay of the AcrH_2_^+•^/TolSQH^•^ RP by stopped-flow measurements as previously described [38]. Rapid mixing of an excess of AcrH_2_ with TolSQ in acetonitrile (MeCN) in the presence of HClO_4_ results in appearance of a transient absorption band at *λ* = 640 nm attributed to formation of AcrH_2_^+•^ species, the decay of which corresponds to both deprotonation and disproportionation of this species [38]. Figure 6 illustrates a diagram of the reaction mechanism. No MFE is evident in the kinetics of this decay at 25°C (illustrated by differences in kinetic traces by the application of a 59.5 mT MF in figure 7 as an example). Similar results were obtained from applying MFs between 11.1 and 163.1 mT (electronic supplementary material, figure S16).

The measurement of significant MFEs, even at moderate field magnitudes, often requires conditions that extend the lifetime of the RP and therefore increases the probability of recombination [33]. We therefore repeated the experiments both at lower temperatures (4°C) and in a more viscous solvent (90% v/v cyclohexanol/MeCN) to limit RP diffusion. However, we were still unable to detect MFEs under these conditions (data not shown). The TolSQH^•^ radical can also undergo disproportionation (figure 6), and does so very rapidly [38]. This system therefore serves as another illustration of rapid quenching of one or more radicals of a pair resulting in negligible recombination. Without significant recombination in this type of RP system, the reaction is no longer spin-selective, and the existence of MFs becomes incidental.

### 3.4. The oxidative half-reaction of pyranose 2-oxidase from *Trametes multicolour*

The enzymatic reaction between reduced flavins and molecular oxygen is involved in redox metabolism, homeostasis, the production of signalling molecules such as H_2_O_2_ and the generation of reactive oxygen species [62]. The existence of RP intermediates in the mechanism of flavin reoxidation (along the lines of that illustrated in figure 1*c*,*d*) has long been proposed [63], the details of which are now being elucidated in enzymes such as P2O from *T. multicolour*. In P2O, electron transfer from fully reduced FADH^−^ to molecular oxygen (a ground-state triplet), coupled with a proton from a conserved histidine, generates a triplet-born neutral flavin semiquinone/HOO**^•^** RP. This must then undergo spin-state mixing to the singlet RP in order to form the C4a-hydroperoxyflavin intermediate (not shown in figure 1). H_2_O_2_ elimination then proceeds to result in fully oxidized FAD [39,64]. Not only is this reaction intrinsically spin-dependent, but it is also analogous to a proposed molecular mechanism for cryptochrome-dependent magnetoreception in animals [65].

**Figure 8.**
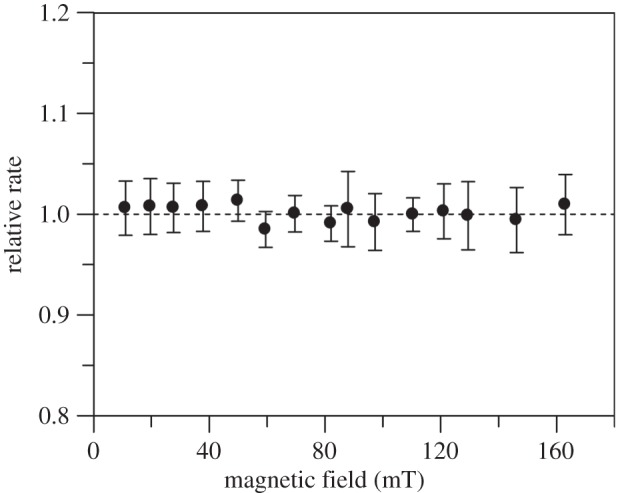
MF dependence of the kinetics of flavin oxidation in P2O from *Trametes multicolor*. Relative rates are calculated for the formation of the C4a-hydroperoxyflavin intermediate in the oxidative-half-reaction of P2O. The data points are the mean of 12 data acquisition pairs (MF_off_ and MF_on_), and the error bars represent the standard deviation for the each calculated relative rate. From the kinetic analysis, *k*_obs1_ is the rate of formation of the C4a-hydroperoxyflavin intermediate (electronic supplementary material, figure S17).

Biphasic stopped-flow transients were recorded at 395 nm according to Sucharitakul *et al*. [39] that represent the formation and decay of the C4a-hydroperoxyflavin intermediate in the oxidative half-reaction of P2O. No significant MFEs were observed under these conditions (figure 8). Further details, including kinetic traces and difference kinetic traces, are in the electronic supplementary material, figures S17 and S18. Theoretical studies suggest that RPs where one unpaired spin is centred on oxygen are unlikely to show MF sensitivity owing to fast relaxation competing with spin-state mixing, especially under weak MF conditions [66]. Recent examples where MFEs have been reported in small inorganic radicals (NO^•^ and O_2_^–•^), applied field magnitudes tend to be in the range of 4.7–18 T, which modulate *S* – *T*_0_ mixing via the Δ*g* mechanism [67,68]. Such fields are 5–6 orders of magnitude stronger than those typically encountered in our environment.

## 4. Conclusion

We have systematically investigated a variety of flavin-dependent redox enzymes for magnetically sensitive reaction kinetics. Despite the possibility of various RP intermediates (figure 1), no MFEs have been observed in the reaction kinetics of the enzymes investigated. It is possible that in each case ‘hydride transfer’ proceeds via a concerted mechanism with no RP intermediates. However, we have demonstrated here (e.g. stepwise hydride transfer between AcrH_2_ and TolSQH and reoxidation of flavin in P2O) and previously (substrate-triggered Co–C bond homolysis in coenzyme B_12_ by EAL) [19] that even if RP intermediates are known to exist they are not sufficient for magnetic sensitivity. Thus, although hydride transfer might be concerted, an absence of observed MFEs is not diagnostic of an absence of RPs. We are confident that MFEs in reaction kinetics are detectable using the apparatus employed in these studies [17,37]. Therefore, in the light of these results, we should reconsider the likelihood of magnetic sensitivity as a result of the RPM occurring in biology. For a RP reaction to be sensitive to MFs, a number of conditions must be met. For example (and this list is by no means exhaustive), the radicals must have time to separate in space, and the S and T spin states must have time to coherently interconvert. However, this process must be rapid compared with incoherent relaxation processes, a fine balance in the context of sensitivity to weak (∼µT) MFs. Additionally, relaxation represents a significant problem in the context of RPs with an oxygen-centred radical. The reaction must also be spin-selective, where at least one reactive fate must be exclusive to either the S or T spin state. Even if a single reactive step in an enzyme system were to fulfil these and other necessary criteria, its biological function would have to be significantly impacted by this. The spin chemistry must therefore be rate limiting, or result in a significant increase in the local concentration of free radicals. It has been recently proposed that even if MFEs were to be observed *in vitro* they might not persist *in vivo* owing to the buffering effects of homeostasis; unless, of course, the system had evolved for them to do so [69]. It appears, therefore, that although radicals are widespread in biology, the conditions for MF sensitivity appear not to be.

## Supplementary Material

Supplementary Material
