# Supplementary material for: Magnetic field effects as a result of the radical pair mechanism are unlikely in redox enzymes
Source: J R Soc Interface. 2015 Feb 6;12(103):20141155. doi: 10.1098/rsif.2014.1155 (PMC4305418; doi:10.1098/rsif.2014.1155)
Supplement: Supplementary Material [file rsif20141155supp1.pdf]

Supplementary Material

Experimental Details

Plasmids, protein expression and purification

Human fibroblast CPR (soluble CPR, lacking the N-terminal membrane-anchoring region) and its FAD domain were expressed in *E. coli* strain BL21(DE3)pLysS from pET-15b plasmid constructs available in our laboratory and purified as described previously (1, 2, 3).

The calmodulin-bound rat nNOS reductase was expressed (from the pCRNNRC plasmid) in *E. coli* JM109 grown in Terrific Broth and purified as reported previously (4). The rat FAD/NADPH domain of the rat nNOS (amino acid residues 987–1463) was expressed in *E. coli* BL21 (DE3) from its plasmid construct in pET11a vector and purified as described previously (5).

Human apoptosis-inducing factor 1 (AIF1) gene encoding the human Δ1-20 AIF1 was commercially synthesised and subcloned into the expression vector pET-28b. The enzyme with histidine tags at both the N-terminus and C-terminus was overexpressed in *E. coli* strain BL21 (DE3) and purified according to Ye et al., 2002 (6).

Human apoptosis inducing factor 2, AIF-M2 (also known as: AMID, apoptosis-inducing factor-homologous mitochondrion-associated inducer of death; or PRG3, p53-responsive gene 3) was expressed in *E. coli* strain HMS174 (DE3) from plasmid pET-15b AIF-M2 previously constructed in our laboratory and purified as described previously (7).

Human cyt b_5 reductase gene was synthesised and cloned into pET-15b vector. The recombinant protein with a N-terminal His-Tag was expressed in *E. coli* BL21 (DE3), grown in 2xYT medium overnight at 20°C after induction using 0.6 mM isopropyl β-D-1-thiogalactopyranoside (IPTG). The enzyme was purified by affinity chromatography using

Ni-IDA resin and kept in 50 mM Tris-HCl buffer, pH 8.0 that contains 20% (v/v) glycerol and 200 mM NaCl.

Human Δ1-30 truncated DHOD gene was synthesised and subcloned into pET-19b vector and the protein was expressed in *E. coli* BL21 (DE3) grown in 4xYT medium and purified as previously reported (8).

Pyranose 2-oxidase (P2O) from *Trametes multicolor* was produced and purified as previously described (9).

All enzymes produced were analysed using UV-visible absorption spectroscopy (*figure S1*) and assessed for purity by SDS-PAGE (*figure S2*). The identity of the enzymes produced by commercial gene synthesis was further confirmed by MALDI within the mass spectrometry facility at the Manchester Institute of Biotechnology, The University of Manchester, UK.

Stopped-flow measurements of 'hydride' transfer

Rapid mixing stopped-flow spectroscopy studies were performed under anaerobic conditions to measure the 'hydride' transfer for the targeted enzymes. Experimental details are as follows:

Stopped-flow measurements of 'hydride' transfer in diflavin oxidoreductases

Human CPR: studies of flavin reduction in the human soluble full length CPR and its FAD domain were performed as previously reported (10). Absorption transients were collected at 450 nm and 600 nm to monitor the reduction of full length enzyme (10 μM) by NADPH (200 μM) in 50 mM potassium phosphate buffer, pH 7.0 at 25 °C. Data are described by a double exponential expression yielding k_{obs1} and k_{obs2} as described previously (10). k_{obs1} reports on the rate of first 'hydride' transfer and internal (interflavin) electron transfer to form the blue di-semiquinone ($FADH^{\bullet}$ /$FMNH^{\bullet}$) state while k_{obs2} represents the rate of formation of the four-electron reduced species (second 'hydride' transfer) (10). For the FAD domain of human

CPR, the reduction of the FAD-bound enzyme (20 μM) by NADPH (400 μM) at 450 nm was recorded also in 50 mM phosphate buffer, pH 7.0. Owing to the rapid kinetics of this reaction, data acquisition was performed in this study at 10 °C. The data were fitted using a double exponential expression (*figure S6*) as described previously (10).

Calmodulin-bound rat nNOS reductase: the 'hydride' transfer was monitored at 458 nm for the reduction of the full length enzyme (10 μM) by NADPH (200 μM) in 50 mM Tris-HCl buffer, 1 mM $CaCl_2$, 10% (v/v) glycerol, pH 7.4 at 25 °C as previously performed (4). 'Hydride' transfer in the FAD domain of rat nNOS reductase (20 μM) was also investigated and the absorption change accompanying flavin reduction was recorded at 454 nm following rapid mixing with NADPH (400 μM) in 50 mM Tris-HCl, 1 mM $CaCl_2$, 10% (v/v) glycerol, pH 7.4 at 25 °C as described previously (5). MFEs were analysed by difference traces (subtracting – MF data from +MF data) due to complexity of data fitting in the full-length enzyme and the FAD domain.

Stopped-flow measurements of 'hydride' transfer in single-site (one flavin) enzymes

Human AIF1: the 'hydride' transfer reaction was monitored by recording the absorbance change at 452 nm following the reduction of enzyme (20 μM) by NADH (10 mM) in 50 mM HEPES buffer, 100 mM NaCl, 1 mM EDTA, pH 8.0 at 25 °C. The data were fitted by a double exponential expression (*figure S9*).

Human AMID: absorption transients were collected at 430 nm to record the 'hydride' transfer during the reduction of the enzyme-bound 6-hydroxy-FAD cofactor for the reaction of enzyme (15 μM) with NADPH (1 mM) in 50 mM potassium phosphate buffer, 300 mM KCl, pH 8.00 at 25 °C. MFEs were analysed by difference traces subtracting – MF data from +MF data) due to complexity of kinetic transients.

Human cyt b_5 reductase: the 'hydride' transfer reaction was recorded by rapid mixing of enzyme (20 μM) with NADH (0.5 mM) and the change of absorbance was monitored at 462 nm in 20 mM Tris-HCl buffer, 100 mM NaCl, 10% (v/v) glycerol, pH 8.0 at 10 °C. The data were fitted by a single exponential expression (*figure S11*).

Human DHOD: the 'hydride' transfer was monitored by following the absorbance at 470 nm during the reduction of DHODH (15 μM) by L-dihydroorotate (2 mM) in 100 mM Tris-HCl buffer, 300 mM NaCl, 10% (v/v) glycerol, pH 8.0 at 4 °C. Data were fitted to a double exponential expression (*figure S13*) as previously described (11).

Stopped-flow studies of the sequential 'hydride' transfer in the reduction of TolSQ by $AcrH_2$

Formation and decay of the transient radical cation ($AcrH_2^{\bullet+}$) formed in the electron transfer reaction from $AcrH_2$ (6 mM) to TolSQ (0.46 mM) in acetonitrile (MeCN) that contains 49 mM $HClO_4$ was monitored under anaerobic conditions at 640 nm by stopped-flow measurements as previously reported (12, 13). The formation and decay of the $AcrH_2^{\bullet+}$ species was monitored at 25 °C. The experiment was further conducted in a relatively viscous solvent 90% (v/v) cyclohexanol/MeCN in presence of $HClO_4$.

Stopped-flow measurements of the oxidative-half reaction of P2O from *Trametes multicolour*

The formation and decay of the C4a-hydroperoxyflavin intermediate formed during the oxidative-half reaction of glucose-reduced enzyme by O_2 enzyme was monitored at 395 nm after mixing the reduced enzyme (22 μM-after mixing) with a buffer solution saturated with O_2 (0.96 mM-after mixing) in 50 mM sodium phosphate buffer , pH 7.0 at 4 °C. Data was fitted to double exponential fit (figure S17) as described previously (14).

Figures

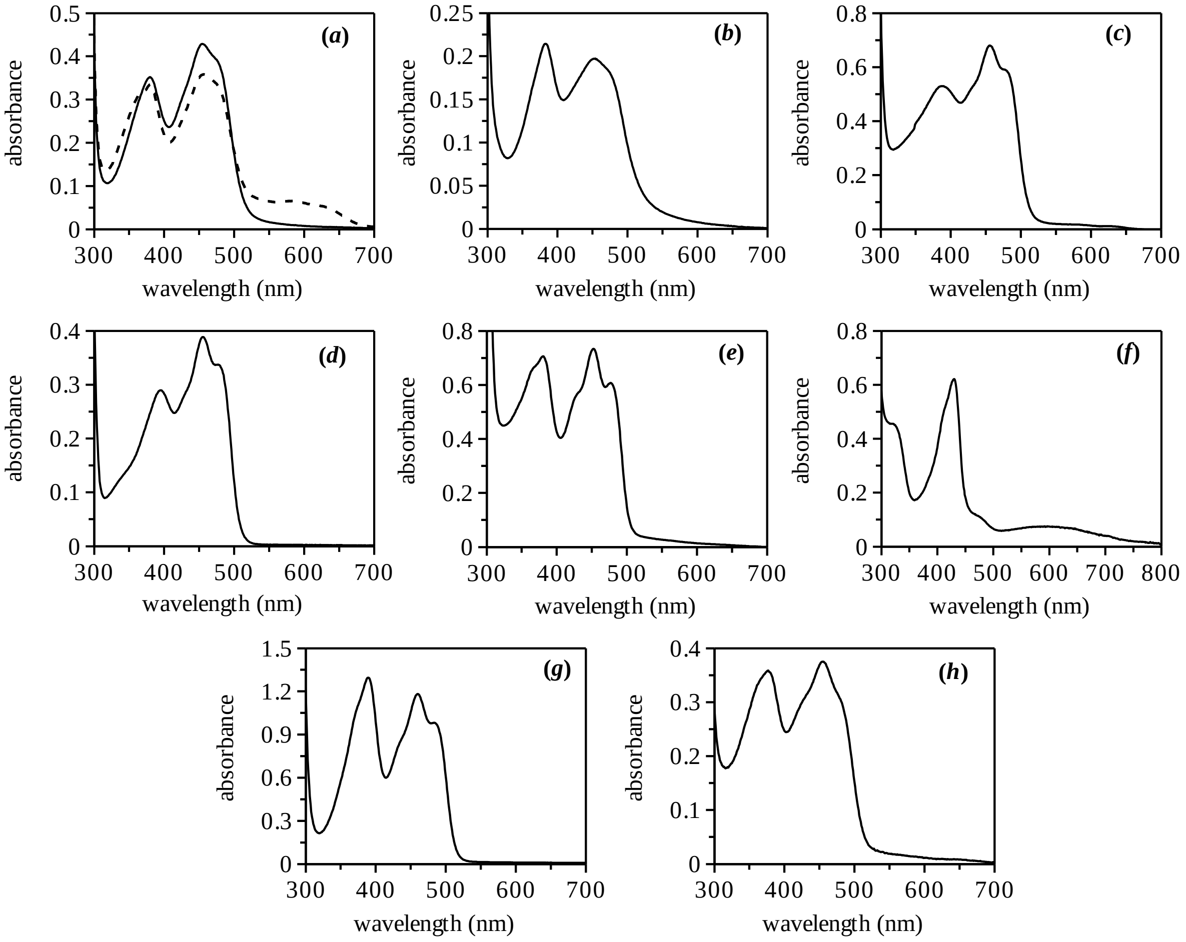

***Figure S1*. UV-visible absorption spectra of the purified enzymes at 25 °C.** (*a*): Human CPR (20 μM) in 50 mM potassium phosphate buffer, pH 7.0, solid line is the spectrum of the oxidised enzyme and dashed line is the spectrum of the as-purified enzymes, (*b*): Human FAD domain of CPR (18 μM) in 50 mM potassium phosphate buffer, pH 7.0, (*c*): Rat nNOS reductase domain (30 μM) in 50 mM Tris buffer, 10% (v/v) glycerol, 1 mM $CaCl_2$, pH 7.4, (*d*): Rat FAD domain of nNOS (38 μM) in 50 mM Tris buffer, 10% (v/v) glycerol, 1 mM $CaCl_2$, pH 7.4, (*e*): Human AIF1 (55 μM) in 50 mM HEPES buffer, 100 mM NaCl, 10% (v/v) glycerol, pH 8.0, (*f*): Human AIF-M2 (24 μM) in 50 mM potassium phosphate buffer, 300 mM KCl, pH 8.0, (*g*): Human cyt b_5 reductase (110 μM) in 20 mM Tris buffer, 100 mM NaCl, 10% (v/v) glycerol, pH 8.0 and (*h*): Human DHODH (33 μM) in 10 mM Tris buffer, 300 mM NaCl, 10% (v/v) glycerol, pH 8.0,

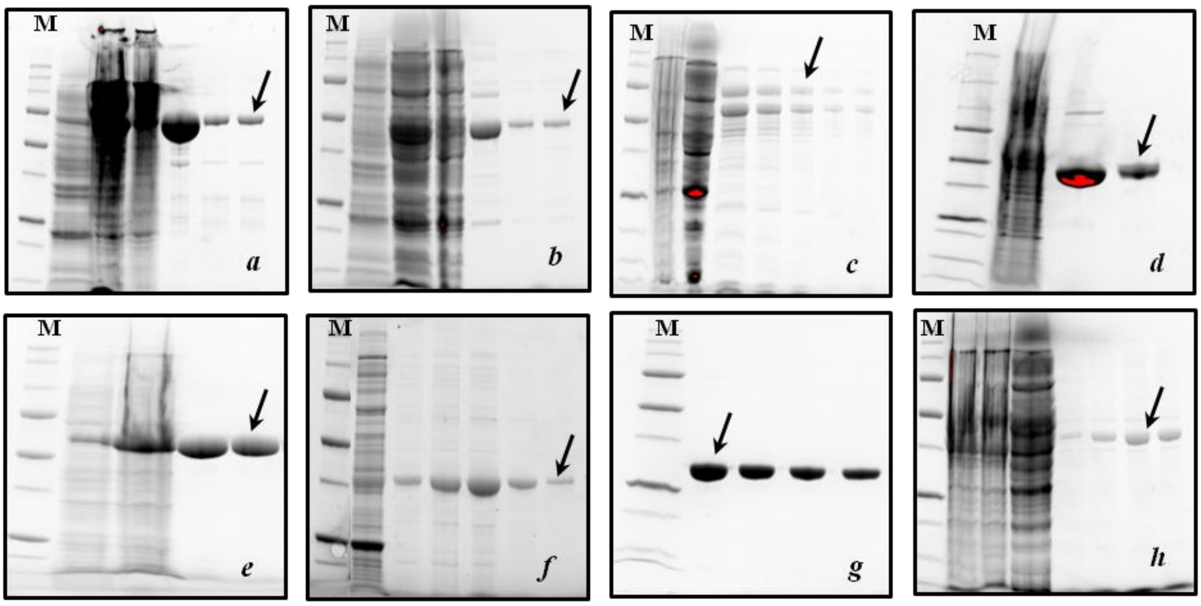

***Figure S2*. SDS PAGE (12%) displaying the purity of various enzymes during various purification steps.** The black arrows indicate the samples that contain pure proteins. M is a protein molecular weight marker of 250, 150, 100, 75, 50, 25, 20, 15, 10 kDa (top to bottom), respectively. (*a*): Human CPR (72 kDa), (*b*): Human FAD domain of CPR (49.7 kDa), (*c*): Rat calmodulin-bound nNOS reductase domain (79 kDa), multiple bands were observed due to cleavage of intact protein during gel electrophoresis, (*d*) Rat FAD domain of nNOS reductase (49 kDa), (*e*): Human AIF1 (57 kDa), (*f*): Human AIF-M2 (42.7 kDa), (*g*): Human cyt b_5 reductase (34 kDa) and (*h*): Human DHODH (43 kDa). Red denotes signal saturation.

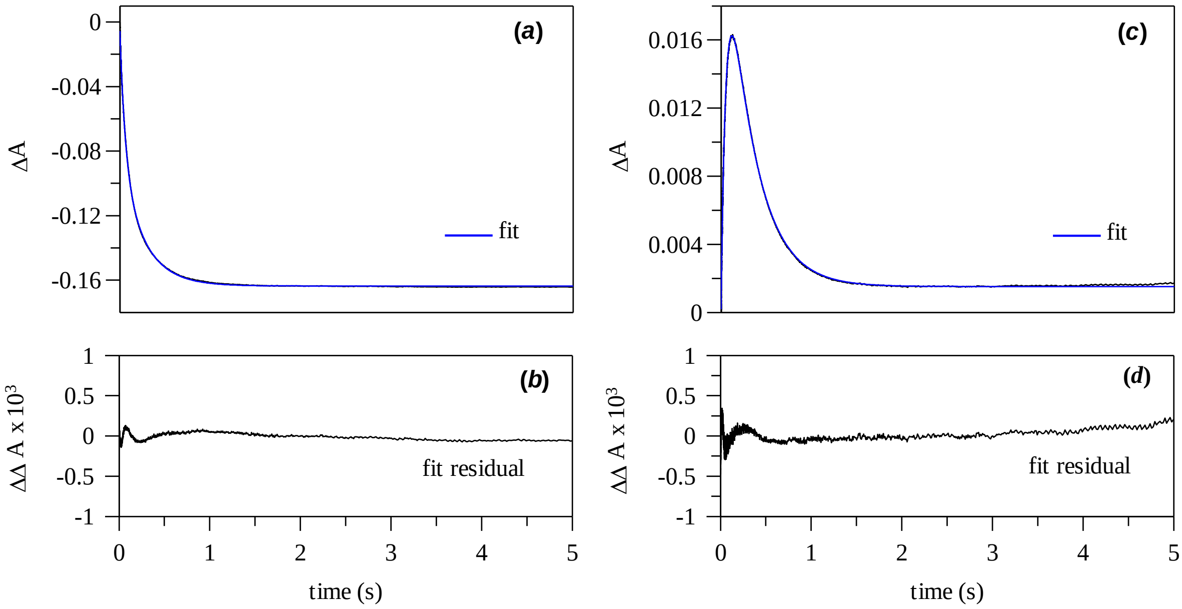

***Figure S3*. Kinetic transients recorded for monitoring 'hydride' transfer reaction of human full length CPR.**

Panel (*a*) Biphasic absorption transient observed at 450 nm; data are described by a double exponential expression (fit in blue) yielding k_{obs1} of 16.6 s^{-1} and k_{obs2} of 3.6 s^{-1}. k_{obs1} reports on the first 'hydride' transfer and internal (interflavin) electron transfer to form the di-semiquinoid ($FADH^{\bullet}/FMNH^{\bullet}$) form of CPR, and k_{obs2} represents the second 'hydride' transfer. Panel (*b*) represents the fit residual.

Panel (*c*) Biphasic absorption transient observed at 600 nm; data are described by a double exponential expression (fit in blue) yielding k_{obs1} of 16.8 s^{-1} and k_{obs2} of 3.46 s^{-1}. k_1 is the rate of formation of the blue di-semiquinoid ($FADH^{\bullet}/FMNH^{\bullet}$) state which represents also the first 'hydride' transfer and k_{obs2} represents the second 'hydride' transfer. Panel (*d*) represents the fit residual.

Conditions: oxidised CPR (10 µM), NADPH (200 µM) in 50 mM potassium phosphate buffer, pH 7.0 at 25 °C under anaerobic conditions.

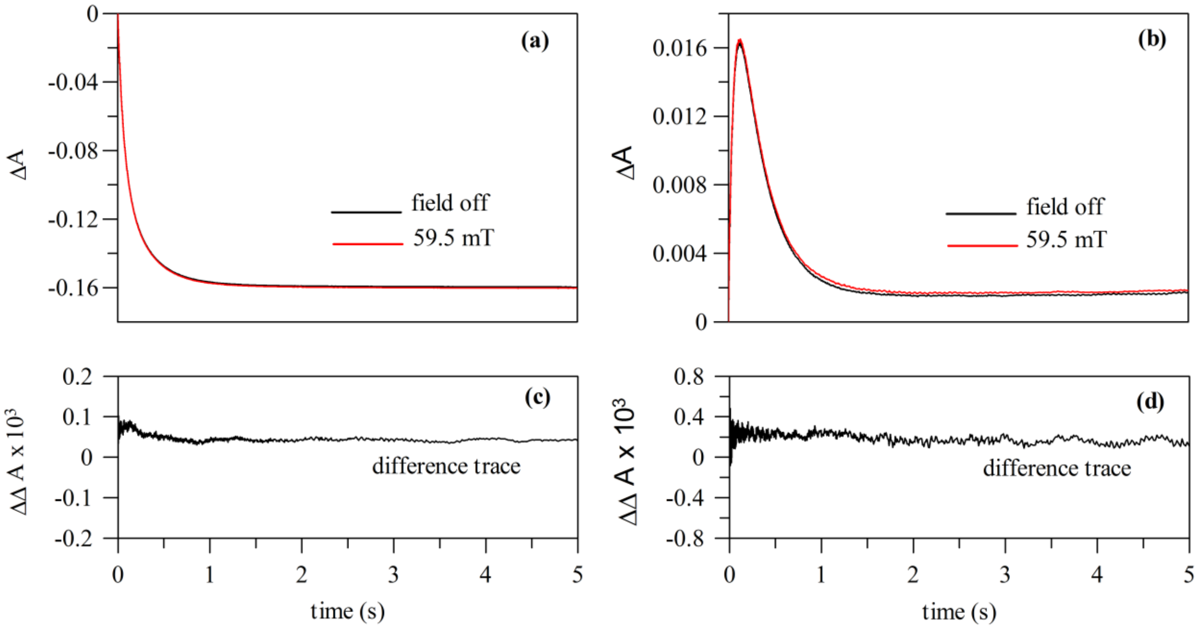

***Figure S4*. Kinetic traces illustrating the influence of magnetic fields on 'hydride' transfer kinetics in human full length soluble CPR.** The traces shown are examples for illustration only.

(*a*) Overlaid traces monitored at 450 nm, acquired at 0 (black) and 59.5 mT (red) and its corresponding difference trace (*b*).

(*c*) Overlaid traces recorded at 600 nm and acquired at 0 (black) and 59.5 mT (red) and its corresponding difference trace (d).

The minor displacement observable in (*a*), (*b*), (*c*) and (*d*) is owing to experimental error and the change in amplitude does not follow a meaningful trend with increasing magnetic field, much like the lack of trend observed for the relative rates (figure 2*a*).

Conditions: oxidised CPR (10 μM), NADPH (200 μM) in 50 mM potassium phosphate buffer, pH 7.0 at 25 °C under anaerobic conditions.

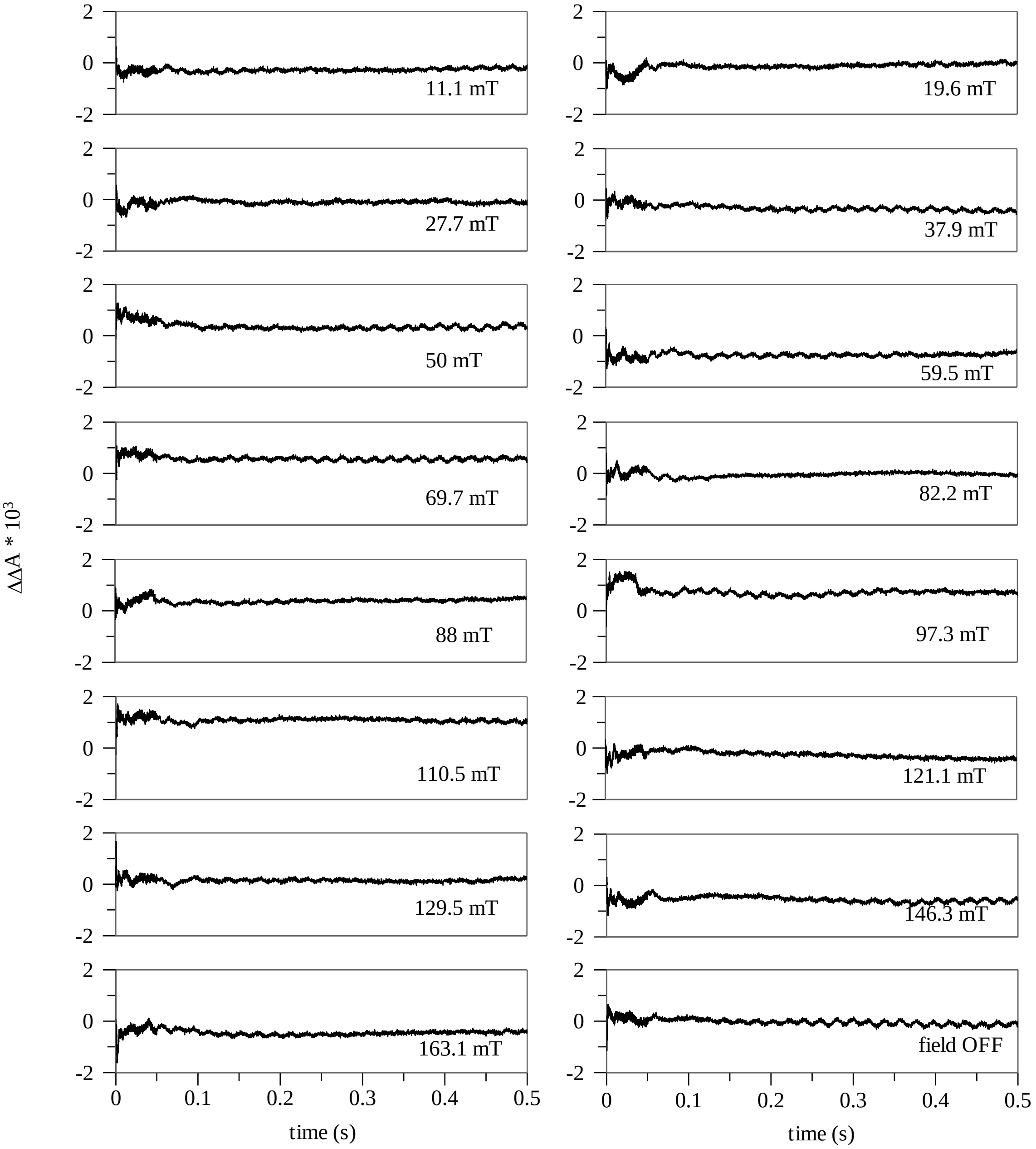

***Figure S5*. Kinetic difference traces illustrating the influence of magnetic fields on 'hydride' transfer kinetics in full-length rat nNOS.**

Each trace is obtained by subtracting the average of 12 field/ON from the average of 12 field/OFF acquisitions at various magnetic field strengths (shown at the bottom of each panel). Any minor displacement observable in each panel is owing to experimental error and the change in amplitude does not follow a meaningful trend with increasing magnetic field. This displacement is also seen in absence of magnetic field (field OFF panel).

Conditions: calmodulin-bound rat nNOS (10 μM), NADPH (200 μM) in 50 mM Tris-HCl buffer, 1 mM $CaCl_2$, 10% (v/v) glycerol, pH 7.4 at 25°C and the change of absorbance was recorded at 458 nm under anaerobic conditions.

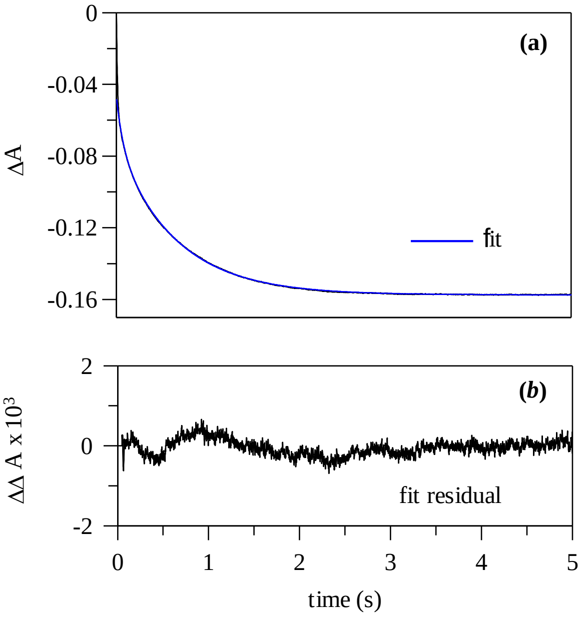

Figure S6. **Kinetic transient recorded for monitoring' hydride' transfer in the FAD domain of human CPR.** (*a*) Biphasic transient observed at 450 nm (black), the fit is displayed in blue, k_{obs1} ~ 37 s^{-1} (formation of NADPH/enzyme charge transfer complex) and k_{obs2} ~ 1.6 s^{-1} (k_{obs2} represents the 'hydride' transfer step (10). Panel (*b*) represents the fit residual.

Conditions: isolated FAD domain of human CPR (20 µM), NADH (400 µM). The change of absorbance was monitored at 450 nm in 50 mM phosphate buffer, pH 7.0 at 10 °C under anaerobic conditions.

N.B. Reduction of the isolated FAD-domain of CR occurs in three kinetically resolvable steps (10). The first step represents a rapid formation of a charge-transfer species between oxidized FAD and NADPH that is very fast to be analysed by stopped-flow measurements. This is followed by isomerization to a second charge-transfer species (k_{obs1} here), and the third step is 'hydride' transfer from NADPH to FAD (k_{obs2} here).

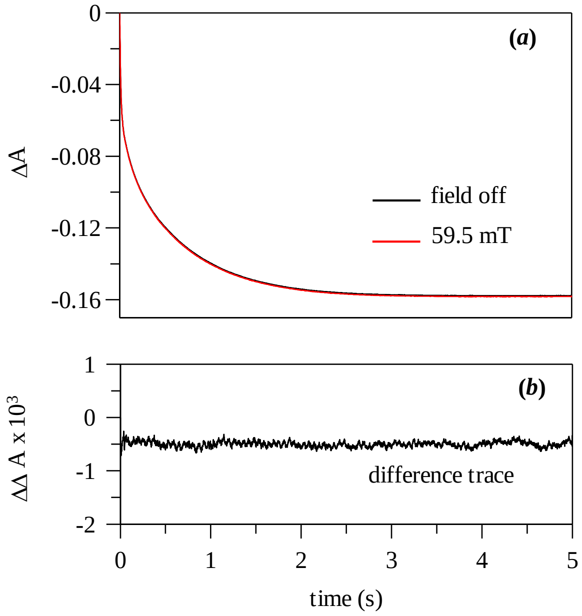

***Figure S7*. The influence of magnetic fields on the 'hydride' transfer kinetics in the isolated FAD domain of human CPR.** (*a*) Overlaid traces recorded at 450 nm, acquired at 0 (black) and 59.5 mT (red) and its corresponding difference trace (*b*). The traces shown are example for illustration only. The minor displacement observable in (*a*) and (*b*) is owing to experimental error and the change in amplitude does not follow a meaningful trend with increasing magnetic field, much like the lack of trend observed for the relative rates (figure 3*a*).

Conditions: isolated FAD domain of human CPR (20 μM), NADH (400 μM). The change of absorbance was monitored at 450 nm in 50 mM phosphate buffer, pH 7.0 at 10 °C under anaerobic conditions.

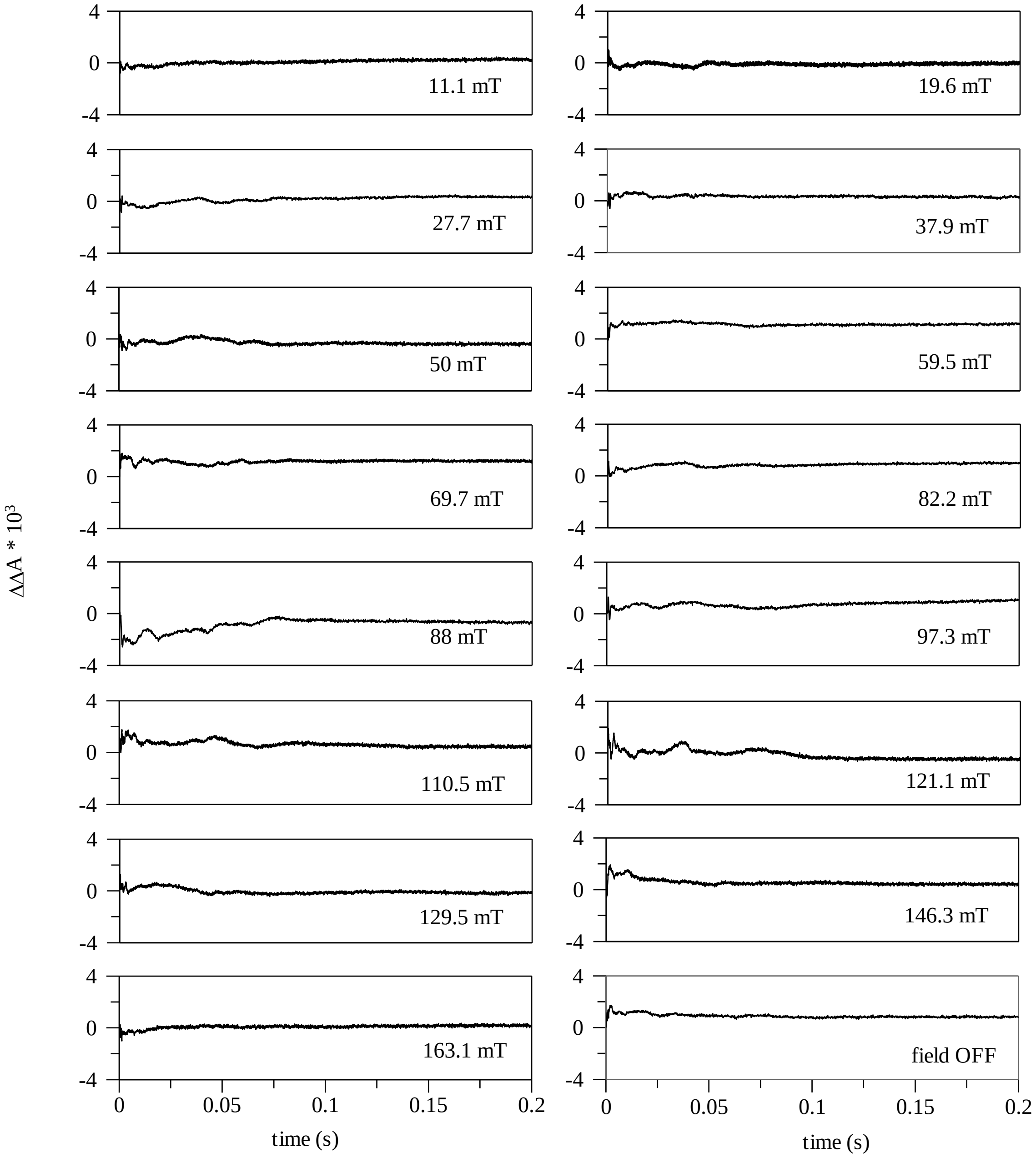

***Figure S8*. Kinetic difference traces illustrating the influence of magnetic fields on 'hydride' transfer kinetics in the isolated FAD domain of rat nNOS.** Each trace is obtained by subtracting the average of 12 field/ON from the average of 12 field/OFF acquisitions at various magnetic field strengths (shown at the bottom of each panel). Any minor displacement observable in all panels is owing to experimental error and the change in amplitude does not follow a meaningful trend with increasing magnetic field. This displacement is also seen in absence of magnetic field (field OFF panel).

Conditions: FAD domain of calmodulin-bound rat nNOS (20 μM), NADPH (400 μM) in 50 mM Tris-HCl buffer, 1 mM $CaCl_2$, 10% (v/v) glycerol, pH 7.4 at 25 °C and the change of absorbance was recorded at 454 nm under anaerobic conditions.

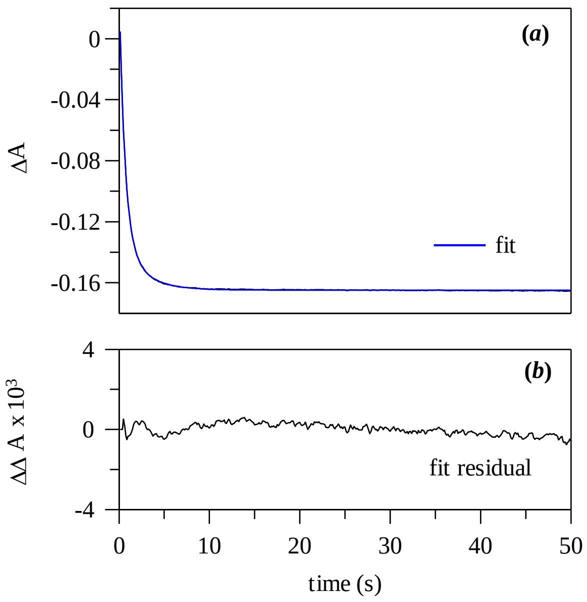

***Figure S9*. Kinetic transient recorded for monitoring 'hydride' transfer of human AIF1.** (*a*) Biphasic transient observed at 452 nm (black) fitted by a double exponential expression, the fit is displayed in blue, k_{obs1} ~ 1.6 s^{-1} ('hydride transfer' rate) and k_2 ~ 0.4 s^{-1} (a slower rate may represent the rate of product release). Panel (*b*) represents the fit residual.

Conditions: AIF1 (20 µM), NADH (10 mM) in 50 mM HEPES buffer, 100 mM NaCl, 1 mM EDTA, pH 8.0 at 25 °C under anaerobic conditions.

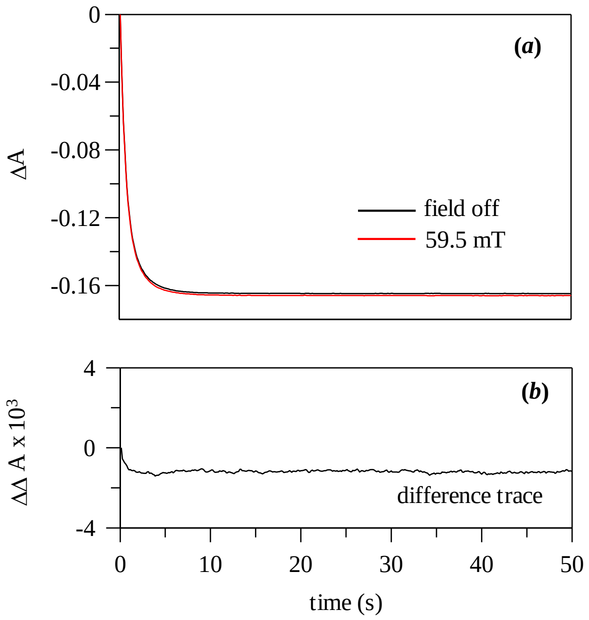

***Figure S10*. Influence of magnetic fields on the 'hydride' transfer kinetics in human AIF1.** (*a*) Overlaid traces recorded at 452 nm, acquired at 0 (black) and 59.5 mT (red) and its corresponding difference trace (*b*). The traces shown are examples for illustration only. The minor displacement observable in (*a*) and (*b*) is owing to experimental error and the change in amplitude does not follow a meaningful trend with increasing magnetic field, much like the lack of trend observed for the relative rates (figure 4*a*).

Conditions: AIF1 (20 μM), NADH (10 mM) in 50 mM HEPES buffer, 100 mM NaCl, 1 mM EDTA, pH 8.0 at 25 °C under anaerobic conditions.

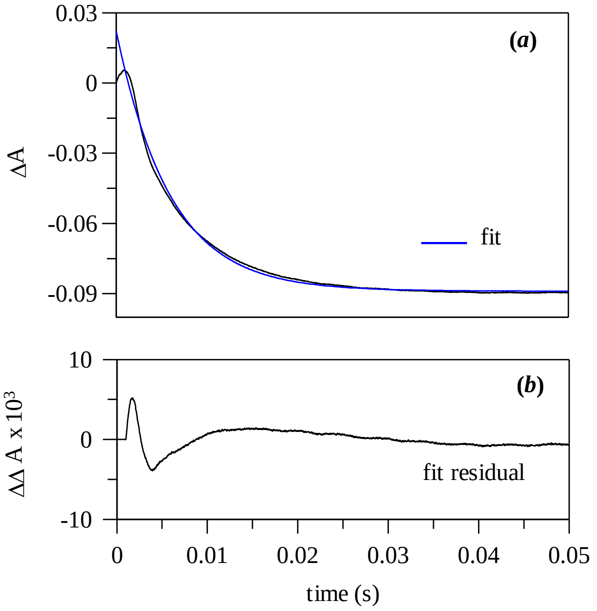

***Figure S11*. Kinetic transient recorded for monitoring 'hydride' transfer of human soluble cyt b_5 reductase.** (*a*) Transient observed at 462 nm (black), the transient is monphasic (fit in blue); 'hydride' transfer rate ($\underline{k_{obs}}$) of 167 s^{-1}. Panel (*b*) represents the fit residual. The artefact recorded at the first few milliseconds lies within the dead time of the stopped-flow apparatus. Conditions: human cyt b_5 reductase (20 μM), NADH (0.5 mM in 20 mM Tris-HCl buffer, 100 mM NaCl, 10% (v/v) glycerol, pH 8.0) at 10 °C under anaerobic conditions.

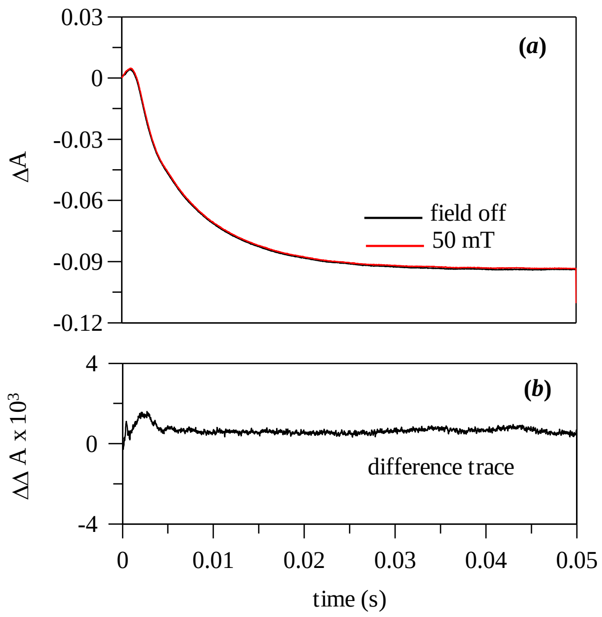

***Figure S12*. The influence of magnetic fields on 'hydride' transfer kinetics in human soluble cyt *b*$_5$ reductase.** (*a*) Overlaid traces recorded at 462 nm, acquired at 0 (black) and 59.5 mT (red) and its corresponding difference trace (*b*). The traces shown are example for illustration only. The increase in absorbance recorded at the first few milliseconds lies within the dead time of the stopped-flow apparatus. The minor displacement observable in (*a*) and (*b*) is owing to experimental error and the change in amplitude does not follow a meaningful trend with increasing magnetic field, much like the lack of trend observed for the relative rates (figure 4*b*).

Conditions: human cyt *b*$_5$ reductase (20 μM), NADH (0.5 mM in 20 mM Tris-HCl buffer, 100 mM NaCl, 10% (v/v) glycerol, pH 8.0) at 10 °C under anaerobic conditions.

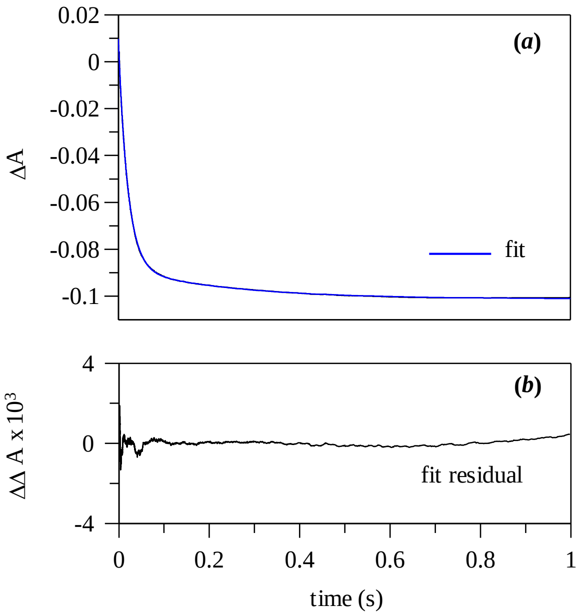

***Figure S13*. Kinetic trace recorded for monitoring 'hydride' transfer of human DHODH.** (*a*) A Biphasic transient observed at 470 nm (black) and its fit (in blue), the 'hydride' transfer rate, k_{obs1} of 49 s^{-1} with 0.09 amplitude change; k_{obs2} is a slower rate of 4.17 s^{-1} with 0.01 amplitude change and could be the rate of orotate dissociation (11). Panel (*b*) is the fit residual.

Conditions: DHODH (15 μM), L-dihydroorotate (2 mM), 100 mM Tris-HCl buffer, 300 mM NaCl, 10% (v/v) glycerol, pH 8.0 at 4 °C and the change of absorbance was monitored under anaerobic conditions.

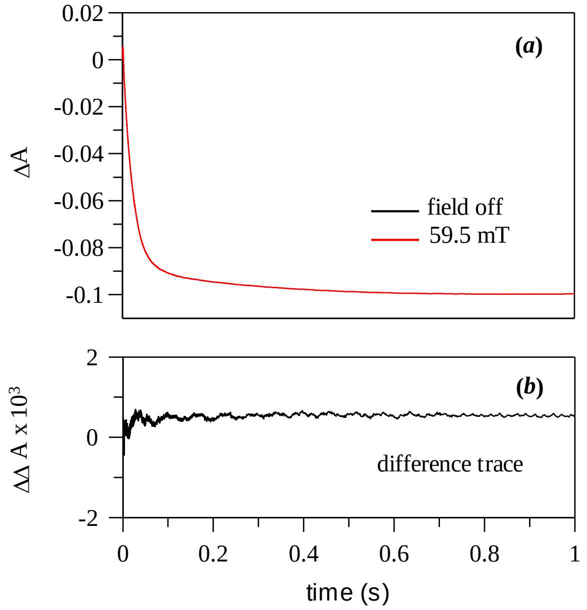

***Figure S14*. The influence of magnetic fields on 'hydride' transfer kinetics in human DHODH.** (*a*) Overlaid traces collected at 470 nm, acquired at 0 (black) and 59.5 mT (red) and its corresponding difference trace (*b*). The traces shown are examples for illustration only. The minor displacement observable in (*a*) and (*b*) is owing to experimental error and the change in amplitude does not follow a meaningful trend with increasing magnetic field, much like the lack of trend observed for the relative rates (figure 4*c*).

Conditions: DHODH (15 µM), L-dihydroorotate (2 mM), 100 mM Tris-HCl buffer, 300 mM NaCl, 10% (v/v) glycerol, pH 8.0 at 4 °C and the change of absorbance was monitored under anaerobic conditions.

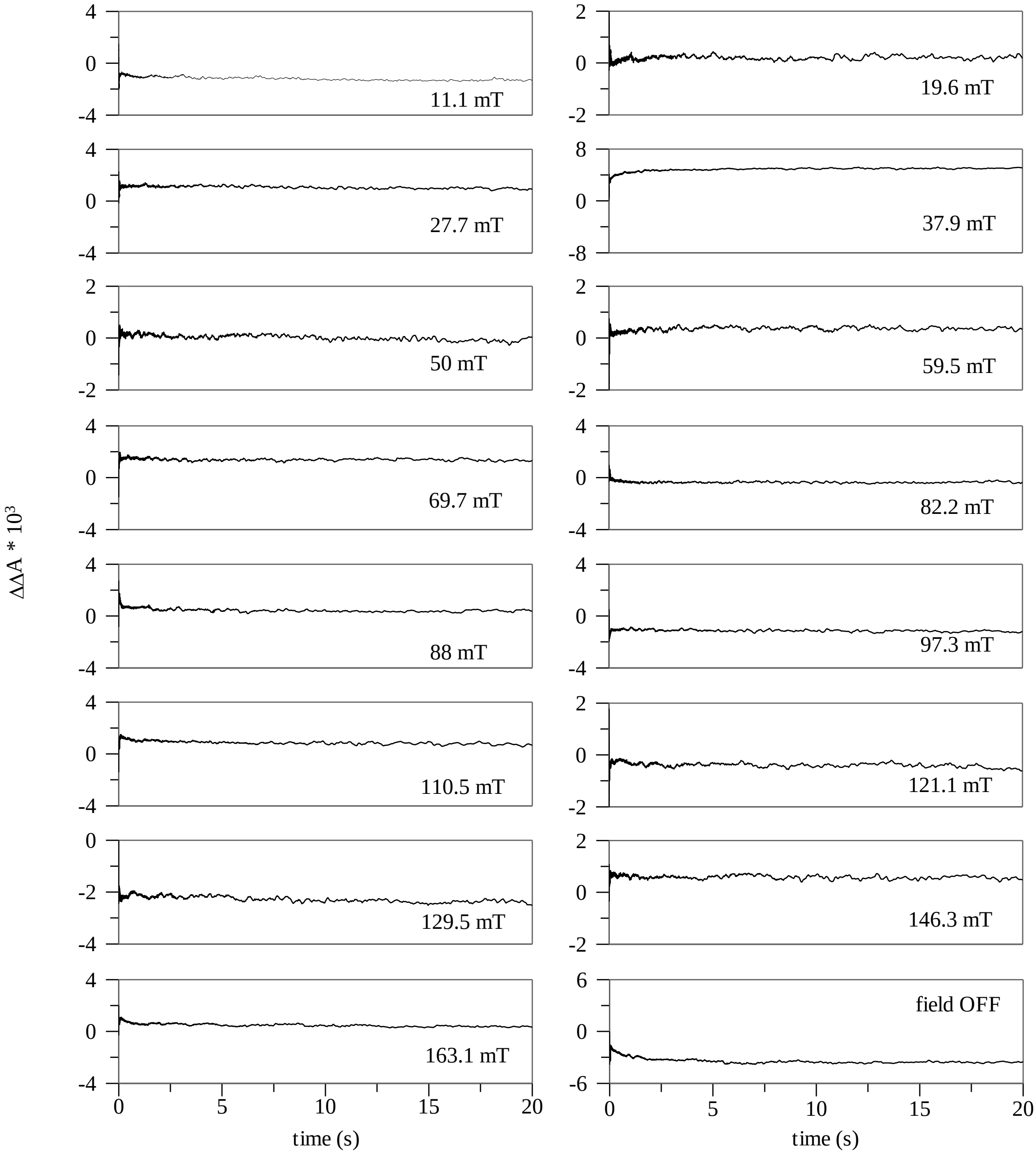

***Figure S15*. Kinetic difference traces illustrating the influence of magnetic fields on 'hydride' transfer kinetics in human AMID.** Each trace is obtained by subtracting the average of 12 field/ON from the average of 12 field/OFF acquisitions at various magnetic field strengths (shown at the bottom of each panel). Any minor displacement observable in all panels is owing to experimental error and the change in amplitude does not follow a meaningful trend with increasing magnetic field. This displacement is also seen in absence of magnetic field (field OFF panel).

Conditions: AMID (15μM), NADPH (1 mM) in 50 mM potassium phosphate buffer, 300 mM KCl, pH 8.00 at 25 °C and the' hydride' transfer reaction was followed by monitoring the flavin reduction at 430 nm under anaerobic conditions.

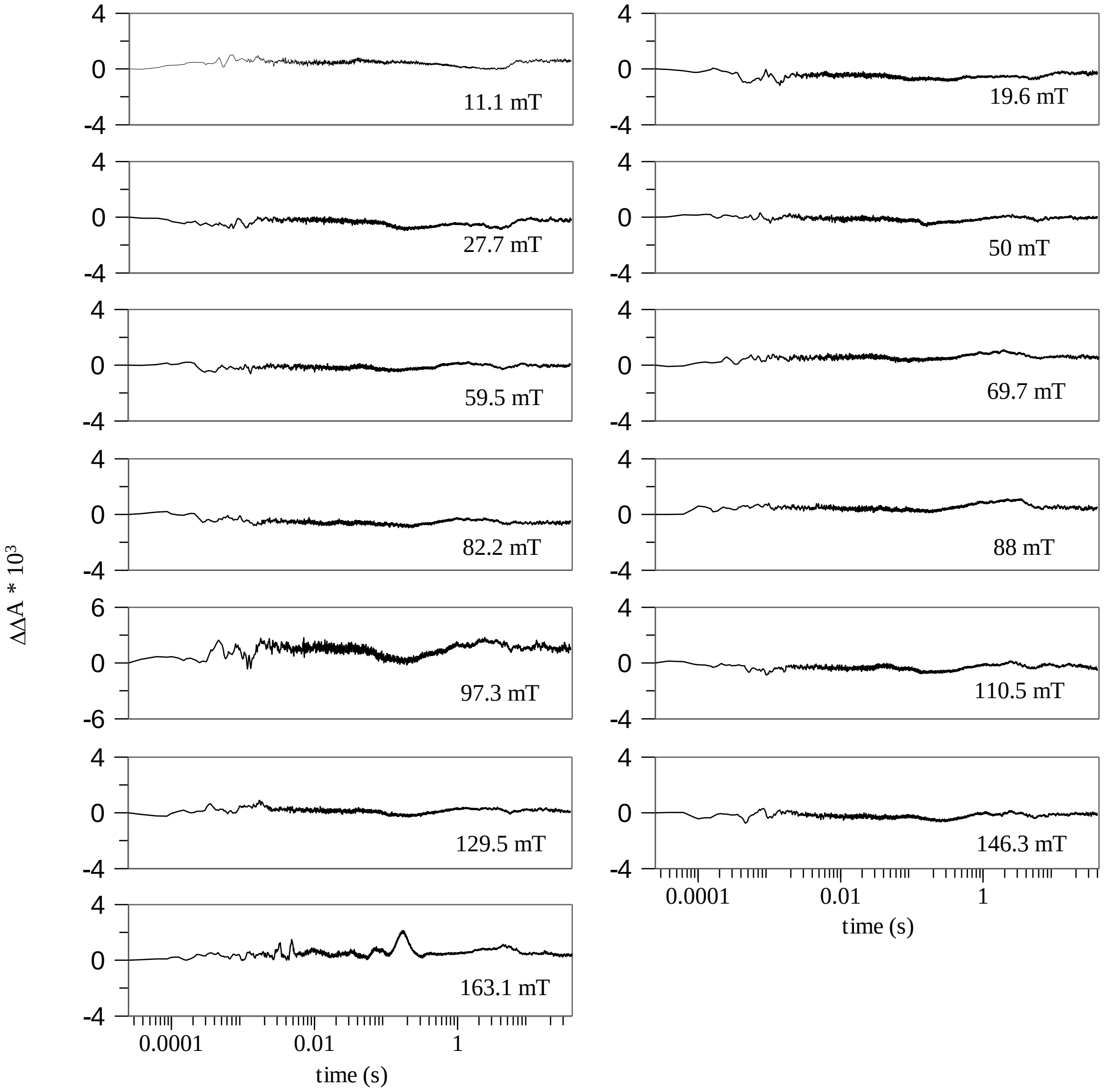

***Figure S16*. Kinetic difference traces illustrating the influence of magnetic fields during the reduction of TolSQ by $AcrH_2$.** Each trace is obtained by subtracting the average of 12 field/ON from the average of 12 field/OFF acquisitions at various magnetic field strengths (shown at the bottom of each panel). The traces record the formation and decay of the $AcrH_2^{+\bullet}$ species monitored at 640 nm in a logarithmic time scale. Any minor displacement observable in all panels is owing to experimental error and the change in amplitude does not follow a meaningful trend with increasing magnetic field.

Conditions: $AcrH_2$ (6 mM), ToLSQ (0.46 mM) in the presence of 49 mM perchloric acid in MeCN at 25 ° C under anaerobic conditions.

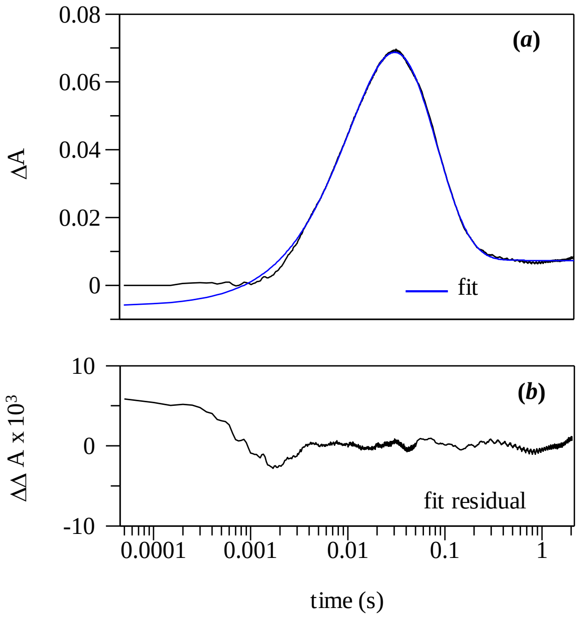

***Figure S17*. Kinetic transient trace recorded for monitoring the oxidative half-reaction of P2O from *Trametes multicolor*.** (*a*) Absorbance trace recorded in a logarithmic time scale at 395 nm (black). The transient is biphasic (fit in blue) showing the C4a-hydroperoxyflavin intermediate formed during the first phase (absorbance increase, k_{obs1} ~ 54 s^{-1}) and decayed during the second phase (absorbance decrease, k_{obs2} ~16.5 s^{-1}), k_2 is also the rate of flavin oxidation (14). Panel (*b*) represents the fit residual.

Conditions: reduced P2O (22 μM), O_2 saturated buffer (0.96 mM O_2), 50 mM sodium phosphate buffer, pH 7.0 at 4 °C under anaerobic conditions.

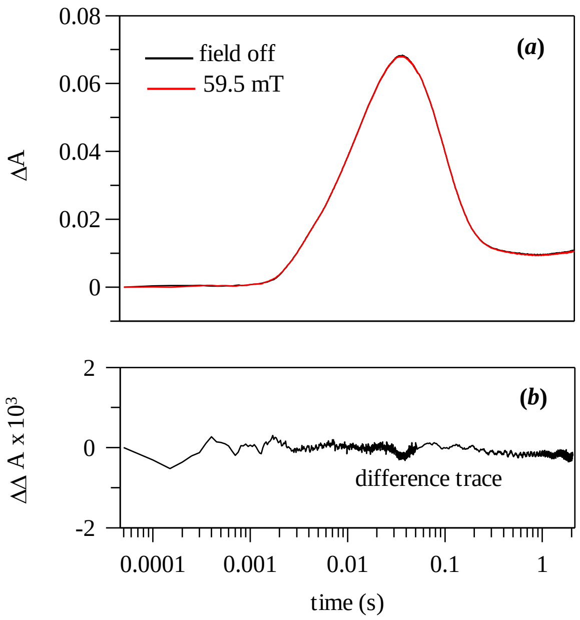

***Figure S18*. The influence of magnetic fields on oxidative half-reaction of P2O from *Trametes multicolor*.** (*a*) Overlaid traces recorded in a logarithmic time scale at 395 nm, acquired at 0 (black) and 50 mT (red) for the oxidative-half reaction of P2O and its corresponding difference trace (*b*). The traces shown are example for illustration only. The minor displacement observable in (*a*) and (*b*) is owing to experimental error and the change in amplitude does not follow a meaningful trend with increasing magnetic field, much like the lack of trend observed for the relative rates (figure 8).

Conditions: reduced P2O (22 μM), O_2 saturated buffer (0.96 mM O_2), 50 mM sodium phosphate buffer, pH 7.0 at 4 °C under anaerobic conditions.

References

1. Smith GCM, Tew DG, Wolf CR. 1994 Dissection of Nadph-Cytochrome P450 Oxidoreductase into Distinct Functional Domains. *P Natl Acad Sci USA* **91**(18), 8710-8714. (Doi:10.1073/pnas.91.18.8710)
2. Zhao Q, Smith G, Modi S, Paine M, Wolf RC, Tew D, et al. 1996 Crystallization and preliminary X-ray diffraction studies of human cytochrome P450 reductase. *J Struct Biol* **116**(2), 320-325. (Doi:10.1006/jsbi.1996.0048)
3. Modi S, Gilham DE, Sutcliffe MJ, Lian LY, Primrose WU, Wolf CR, et al. 1997 1-methyl-4-phenyl-1,2,3,6-tetrahydropyridine as a substrate of cytochrome P450 2D6: Allosteric effects of NADPH-cytochrome P450 reductase. *Biochemistry-Us* **36**(15), 4461-4470. (Doi:10.1021/Bi962633p)
4. Knight K, Scrutton NS. 2002 Stopped-flow kinetic studies of electron transfer in the reductase domain of neuronal nitric oxide synthase: re-evaluation of the kinetic mechanism reveals new enzyme intermediates and variation with cytochrome P450 reductase. *Biochem J* **367**, 19-30. (Doi:10.1042/Bj20020667)
5. Dunford AJ, Marshall KR, Munro AW, Scrutton NS. 2004 Thermodynamic and kinetic analysis of the isolated FAD domain of rat neuronal nitric oxide synthase altered in the region of the FAD shielding residue Phe1395. *Eur J Biochem* **271**(12), 2548-2560. (Doi:10.1111/j.1432-1033.2004.04185.x)
6. Ye H, Cande C, Stephanou NC, Jiang SL, Gurbuxani S, Larochette N, et al. 2002 DNA binding is required for the apoptogenic action of apoptosis inducing factor. *Nat Struct Biol* **9**(9), 680-684. (Doi:10.1038/Nsb836)
7. Marshall KR, Gong M, Wodke L, Lamb JH, Jones DJL, Farmer PB, et al. 2005 The human apoptosis-inducing protein AMID is an oxidoreductase with a modified flavin cofactor and DNA binding activity. *J Biol Chem* **280**(35), 30735-30740. (Doi:10.1074/jbc.M414018200)
8. Neidhardt EA, Punreddy SR, McLean JE, Hedstrom L, Grossman TH. 1999 Expression and characterization of E. coli-produced soluble, functional human dihydroorotate dehydrogenase: a potential target for immunosuppression. *Journal of molecular microbiology and biotechnology* **1**(1), 183-188.
9. Leitner C, Volc J, Haltrich D. 2001 Purification and characterization of pyranose oxidase from the white rot fungus Trametes multicolor. *Appl Environ Microb* **67**(8), 3636-3644. (Doi:10.1128/Aem.67.8.3636-3644.2001)
10. Gutierrez A, Lian LY, Wolf CR, Scrutton NS, Roberts GCK. 2001 Stopped-flow kinetic studies of flavin reduction in human cytochrome P450 reductase and its component domains. *Biochemistry-Us* **40**(7), 1964-1975. (Doi:10.1021/Bi001719m)
11. Fagan RL, Nelson MN, Pagano PM, Palfey BA. 2006 Mechanism of flavin reduction in class 2 dihydroorotate dehydrogenases. *Biochemistry-Us* **45**(50), 14926-14932. (Doi:10.1021/bi060919g)
12. Yuasa J, Yamada S, Fukuzumi S. 2008 Detection of a radical cation of an NADH analogue in two-electron reduction of a protonated p-quinone derivative by an NADH analogue. *Angew Chem Int Edit* **47**(6), 1068-1071. (Doi:10.1002/anie.200704136)
13. Yuasa J, Yamada S, Fukuzumi S. 2008 One-step versus stepwise mechanism in protonated amino acid-promoted electron-transfer reduction of a quinone by electron donors and two-electron reduction by a dihydronicotinamide adenine dinucleotide analogue. Interplay between electron transfer and hydrogen bonding. *J Am Chem Soc* **130**(17), 5808-5820. (Doi:10.1021/Ja8001452)

14. Sucharitakul J, Prongjit M, Haltrich D, Chaiyen P. 2008 Detection of a C4a-hydroperoxyflavin intermediate in the reaction of a flavoprotein oxidase. *Biochemistry-Us* **47**(33), 8485-8490. (Doi:10.1021/Bi801039d)
